# Matrix factorization-based multi-objective ranking–What makes a good university?

**DOI:** 10.1371/journal.pone.0284078

**Published:** 2023-04-13

**Authors:** János Abonyi, Ádám Ipkovich, Gyula Dörgő, Károly Héberger

**Affiliations:** 1 Eötvös Loránd Research Network - University of Pannonia Complex Systems Monitoring Research Group, University of Pannonia, Veszprém, Hungary; 2 Plasma Chemistry Research Group, Institute of Materials and Environmental Chemistry, Research Centre for Natural Sciences, Centre of Excellence, Hungarian Academy of Sciences, Budapest, Hungary; University of Tehran, IRAN, ISLAMIC REPUBLIC OF

## Abstract

Non-negative matrix factorization (NMF) efficiently reduces high dimensionality for *many*-objective ranking problems. In multi-objective optimization, as long as only three or four conflicting viewpoints are present, an optimal solution can be determined by finding the Pareto front. When the number of the objectives increases, the multi-objective problem evolves into a *many*-objective optimization task, where the Pareto front becomes oversaturated. The key idea is that NMF aggregates the objectives so that the Pareto front can be applied, while the Sum of Ranking Differences (SRD) method selects the objectives that have a detrimental effect on the aggregation, and validates the findings. The applicability of the method is illustrated by the ranking of 1176 universities based on 46 variables of the CWTS Leiden Ranking 2020 database. The performance of NMF is compared to principal component analysis (PCA) and sparse non-negative matrix factorization-based solutions. The results illustrate that PCA incorporates negatively correlated objectives into the same principal component. On the contrary, NMF only allows non-negative correlations, which enable the proper use of the Pareto front. With the combination of NMF and SRD, a non-biased ranking of the universities based on 46 criteria is established, where Harvard, Rockefeller and Stanford Universities are determined as the first three. To evaluate the ranking capabilities of the methods, measures based on Relative Entropy (RE) and Hypervolume (HV) are proposed. The results confirm that the sparse NMF method provides the most informative ranking. The results highlight that academic excellence can be improved by decreasing the proportion of unknown open-access publications and short distance collaborations. The proportion of gender indicators barely correlate with scientific impact. More authors, long-distance collaborations, publications that have more scientific impact and citations on average highly influence the university ranking in a positive direction.

## Introduction

The present work is motivated by Einstein’s quote, “Everybody is a genius. But if you judge a fish by its ability to climb a tree, it will live its whole life believing that it is stupid.”, *i.e.* everything should be measured against appropriate standards. This quote inherently exposes the core problem of the present research: the higher the number of objectives (dimensions) is, the more solutions become optimal from a perspective. The selection of the optimal solutions based on three or more objectives is called multi-objective optimization.

In most multi-objective optimization problems, the objectives are often conflicting. Finding the Pareto front can be the key to solving multi-objective optimization problems consisting of two or three objectives as it selects the optimal solutions. Yet, most Pareto-based methods cannot optimize a problem with more than four conflicting objectives [1]. The Pareto dominance performs worse with increasing dimensionality, which results in a *many*-objective optimizations problem (MaOO). The main attribute of the problem is the *curse of dimensionality*.

A possible solution is to use dimensionality reduction to reduce the number of objectives and enable the successful application of Pareto fronts. The principal component analysis (PCA) [2] is a popular dimensionality reduction method. The objectives are incorporated into principal components (PC), where the ones with the least variance are discarded to reduce dimensionality. The PCs incorporate positively and negatively correlating objectives, and the Pareto front selects those solutions that excel in both.

Our research objectives are to develop a general algorithm that is capable of:

- Selecting the best solutions considering as many objectives as possible, and uncovering why they excel.
- Diminishing the *curse of dimensionality*, as high number of objectives result in more solutions becoming part of the set of optimal solutions, the Pareto front.
- Projecting the data to positive objective-space so that the reduced components do not contain negatively correlating objectives. This way, the applicability of the Pareto front remains.
- Assigning weights to a group of similar objectives to overcome subjectivity, while also reducing the impact of reduntant objectives on the objective-space.
- Consistently aggregate and select the most important objectives, improving the correctness and objectivity of the analysis, as the redundant variables may influence the components composition, and alter the weighting.

The main idea is to meet the above-mentioned requirements by applying sparse non-negative matrix factorization (sparse NMF) [3, 4]. The reduced components are constrained to non-negative decision space. In the special case of two or three dimensions, the Pareto front of the “good” objectives can be accessed, providing proper data visualization. Moreover, the weights should be considered for the group, rather than the individual objectives so that the redundant ones may not distort the analysis. The use of dimensionality reduction method may tackle the problem of subjective weights as the PCA has been shown to reduce subjectivity [5]. The NMF [6] is also included in the work for comparison.

The conflicting objectives (or those ranked in reverse) need to be distinguished to understand the underlying structure of the objectives and the ranking outcome. The validation of the dimensionality reduction should ensure that the correlational analysis yields the same as other established methods. The sum of ranking differences (SRD) technique [7–9] performs exceptionally well in validating the NMF as it can qualify the objectives with its randomization test; hence, it can classify the objectives as similar, outlier, as well as having forward-, random- and reverse rankings. The SRD can determine the optimality of an objective by calculating the city block (Manhattan) distances against an ideal reference. Should the SRD values be closer to zero, the objective will resemble the ideal reference more closely. In case of missing ideal reference, one can be generated through the aggregation of data, *i.e.*, maximum, mean, median, minimum of the data. In the present work, the SRD method is employed for validation purposes.

The goal of applying NMF with SRD filtering is to explore the relationships behind the factors and create aggregated variables from which the Pareto front can be formed. NMF provides positively correlating objectives, as opposed to the PCA, therefore, yields a valid Pareto front. The optimal solutions are ranked to determine university ranking based on the purely bibliographic CWTS Leiden Ranking 2020 [10] dataset. In addition, several different techniques (principal component analysis (PCA), NMF, and sparse NMF are compared. SRD method is applied to validate the results.

The novelty of the application of NMF is the following:

- A dataset with many objectives facilitates the oversaturation of non-dominated solutions (the best ones). Therefore, we apply dimensionality reduction so that we can use the Pareto front to select from among the solutions in lower-rank approximation of the objective space. Similarly to the concept of the PCA-PROMETHEE method, we introduce NMF and the sum of ranking differences to validate the results, and overcome the saturation of the front. In contrast, we also include PCA, though, valid Pareto front cannot be placed on the PCA-based reduced solutions as conflicting objectives are aggregated into the same principal component. sparse NMF is added to the analysis to find the dominant objectives and remove the redundant and irrelevant ones to make the analysis more comprehensive.
- The objectives used for ranking the universities are contradictory, conflicting, and their evaluation is not trivial, though dimensionality reduction solves the problem. Even if the PCA provides some insights, the crucial grouping can only be completed by SRD and NMF (or their coupling).
- We present the efficiency of the NMF for the dimensionality reduction of ranking problems, where *many*-objective optimization problems are reverted to multi-objective optimization problems. The solutions in the reduced objective space are capable of hosting valid Pareto fronts, as the *curse of dimensionality* is diminished.
- We use NMF to enforce a non-negativity constraint on the data, therefore, incompatible objectives (with each other) cannot be grouped (unlikely for other PCA-like traditional dimensionality reduction techniques). We cover the low-dimensional projection of the solutions with a Pareto front due to the solely positively aligned objectives.
- We validate the NMF with the SRD method, both yielded a similar outcome (the order of the objectives).
- We employ sparse NMF to remove the redundant objectives, which results in a more balanced weighting, and show, which objectives are imperative to the analysis.
- We use NMF to cluster the indicators and universities. The method detects the cultural differences between nationalities by concentrating the aggregated objective on a component, and cultures may earn different aggregated scores. The reduced dimensionality incorporates almost all aspects of the original objective space and highlights these differences in the rankings.

A complex MaOO problem is the ranking of universities, where various viewpoints need to be considered for a fair ranking. The CWTS Leiden Ranking [10] is a popular and widely known set of university indicators that includes numerous categories in several disciplines. Many other rankings neglect the diverse scientific, open-access, collaboration, and gender indicators. The immense number of objectives in the CWTS Leiden Ranking presents the problems and a realization of a use case for MaOO. A recent survey also suggests that the CWTS Leiden Ranking is one of the best choices for independent university ranking [11].

MaOOs are becoming influential to the world of optimization; by using NMF, a fast and efficient solution is proposed. The NMF is a standard method for implementing decision-making algorithms, and is often used in feature extraction, which can be considered as a MaOO problem [12]. Yet, its utilization in MaOO type *many*-objective university ranking as thoroughly as this was and still is unknown to the authors.

The sparse NMF is provided by the Non-Negative Matrix Factorization Toolbox [13]. All results stated below were implemented in MATLAB, and the resultant codes of the proposed non-negative matrix factorization-based ranking and visualization algorithms are publicly available on Zenodo.

In the following sections, the related works are discussed. Then the methodology is introduced, where the problem of the classical approach is presented. Next, the dimensionality reduction techniques, performance metrics, and the SRD method are described. Lastly, the outcome is discussed and concluding remarks are provided. Supporting information on the Technique for Order of Preference by Similarity to Ideal Solution (TOPSIS) [14] S1 Appendix and the composition of the principal components S2 Appendix can be found in S1 Appendix. A nomenclature (S3 and S4 Appendices) is also provided to help the reader to understand the paper better.

### Related works

A possible solution to the *curse of dimensionality* is the application of multi-criteria decision analysis (MCDA). Most approaches focus on evaluating the objectives through a set of feasible solutions. Many employ aggregation or feature selection. Aggregation evaluates objectives by an aggregation function such as mean or sum, while the feature selection selects the dominant or the most relevant objectives and ranks accordingly. An example commonly employed in MCDA is the Technique for Order of Preference by Similarity to Ideal Solution (TOPSIS) [14], which utilizes aggregation-based ranking by calculating the distance of the solutions from positive and negative ideal references. An alternative approach to MCDA is to extend existing methods, for example, the robust model for TOPSIS to be used in group decision-making [15]. Although fast and efficient, the comprehensive visualization of data and the relationship of objectives remains unsolved. Another example of MCDA methods introduces an automatic ranking method for accurately selecting the best solutions [16].

In the world of MCDA, a consensus between the methods is rare, as the same results are obtained only in a few (somewhat random) cases. [17] arranged 56 available MCDA methods based on their characteristics to a hierarchy and built a framework to support the selection of an MCDA method for a specific decision-making situation. [18] carried out thorough research on the phenomena of ranking inconsistencies, revealing that MCDA methods ranked the data differently. Namely, up to 20% of the initial ranging order was predicted correctly. Lourenço and Lebensztajn proved that by applying the SRD algorithm to the results of MCDA methods, a consensus of eight algorithms could be met [19]. This method is a jack-of-all-trades as validation [20], filtering, non-parametric modeling [21] and multi-criteria decision analysis [22] can be carried out with this one method. In this work, the SRD is used for filtering and validation, as the method is straightforward, popular, highly flexible, and a repeatedly proven validation algorithm.

**Fig 1:**
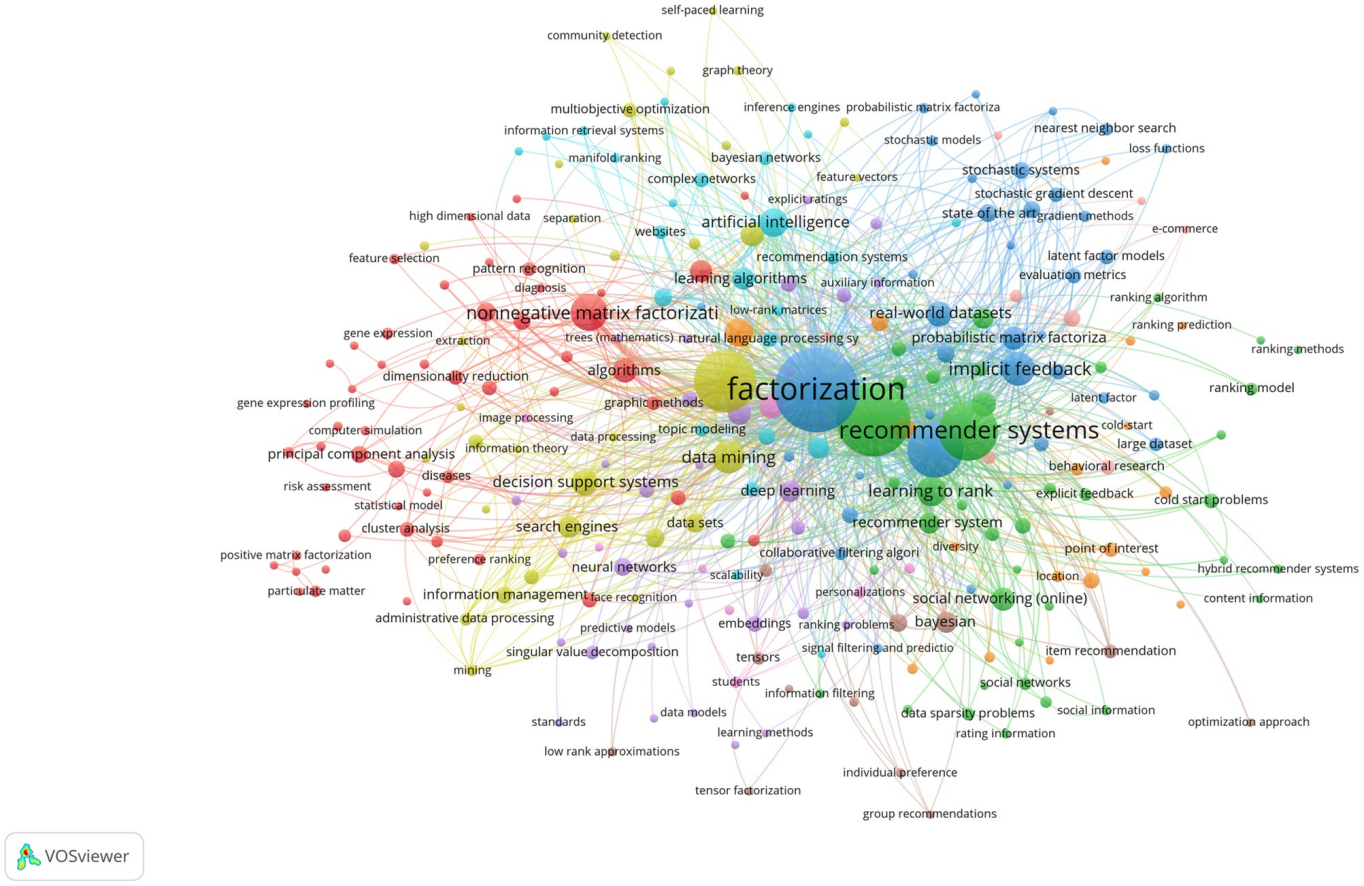
Literature of Matrix factorization and MOO. The figure shows the cooccurrences of keywords in articles from 497 publications queried using Scopus. The search expressions/keywords were matrix factorization and ranking, multi-objective optimization, decision support and multi-objective decision-making. Unique keywords, denoted by the nodes, are connected if they cooccur at least four times. Only publications before the end of December, 2021 are included.

The NMF has a wide range of application possibilities due to its dimensionality reduction features; therefore, an investigation was concluded. Fig 1 demonstrates the clustered version of recent publications. The figure consists of the keywords (denoted by the nodes) of 497 publications queried from Scopus with the search expressions matrix factorization and ranking, multi-objective optimization, decision support and multi-objective, or multi-criteria decision-making. Two nodes are connected if the keywords cooccur in publications more than or equal to four times. The figure can be split into clusters based on the different colors of the nodes, indicating more related publication fields. On the left-hand side, the red group mostly consists of clusters related to NMF. The top (turquoise) clusters are related to artificial intelligence with keywords such as learning algorithms and complex networks. The green clusters slightly to the bottom of the right-hand side represent *recommender system*-related concepts, *e.g.*, social networking and learning to rank. The blue group of clusters represents real-world problems with state-of-the-art stochastic approaches. It can be concluded that matrix factorization is a popular algorithm with vivid applications, even though university ranking cannot be found in the list of frequent keywords.

The main profile of matrix factorization methods is the field of *recommender systems* [23]. An example is social media applications that recommend people based on relationships and preferences [24]. *Recommender systems* also employ a technique called collaborative filtering to extract information from the Internet efficiently. It consists of explicit and implicit ratings; the former depends on what other users prefer, and the latter focuses on user preferences. Collaborative filtering is a popular research topic, as it provides efficient recommender systems, and a possible implementation can be handled by matrix factorization.

[25] provided an example of collaborative filtering: the robust collaborative filtering based on NMF. The amalgamation of matrix factorization and other methods has appeared as the base for *recommender systems*, which improved overall performance and efficiency [26]. Other applications of NMF include face recognition by feature extraction [27] and text clustering [28].

A classic example of MaOO is the ranking of universities, which are inherently complex as these institutions can be ranked according to an endless number of viewpoints, *e.g.*, the fields, research, income, and authorships. It should be noted that the ideal ranking of universities is impossible to establish. In various rankings, the order of universities tends to match at the top and the bottom. Yet, the differences in the “middle” of the ranking depend on the method. Several methods have been applied to determine a fair ranking of the institutions, *e.g.*, Sziklai proposed the Weighted Top Candidate (WTC) algorithm that ranked the university by a novel selection method [29]. Kong *et al.* presented the University Profiling Framework by implementing deep learning and clustering to compare universities [30].

## Materials and methods

**Fig 2:**
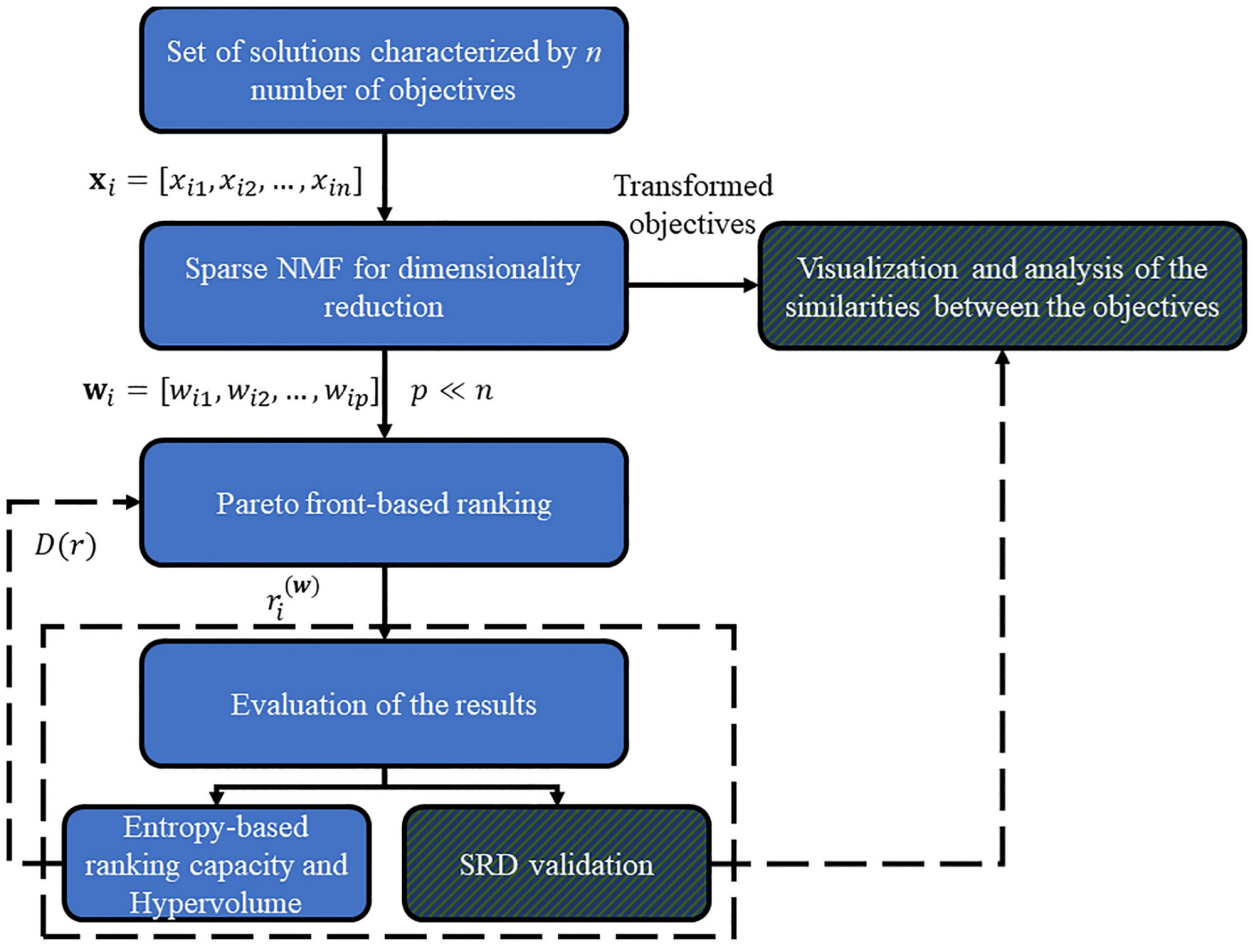
Flowchart of the non-negative matrix factorization-based *many*-objective optimization method. The input of the workflow requires a dataset containing *N* number of solutions (**x**) and *n* objectives. The sparse NMF reduces the objectives to a *p* amount (*p* < < *n*). Information for both the solutions and objectives is attained. The objectives are compared, and the Pareto front is applied to the solutions in the low-rank representation of the objective space (the reduced notation for **x** is denoted by **w**). A solution’s rank is established in the lower-dimensional space as *r*^(**w**)^. The Pareto front is evaluated through the application of Entropy, and Hypervolume (*i.e.*, high saturation and being far from the ideal front may show that the Pareto front is underperforming). The analysis workflow also aims to uncover the relationships between the objectives: the information uncovered from the sparse NMF is verified by Sum of Ranking differences.

During the analysis of high-dimensionality datasets, one must consider the effects of the *curse of dimensionality*. After the data is preprocessed, we use sparse non-negative matrix factorization to decrease the number of objectives while simultaneously removing redundant variables. The importance of the removal concerns the Pareto front, the set of non-dominated solutions. Entropy measures the saturation (*D*(*r*)) of (the effect of the *curse of dimensionality* on) the Pareto front, as adding more objectives increases the number of tied ranks, so the information content disappears, or at least diminishes. If the entropy of the Pareto front is low, then the number of firsts become too numerous. Hypervolume calculates how close the current front is located to a (maximal performance) reference point so that the quality of the front is evaluated. Moreover, the PCA, NMF and sparse NMF provide information on the relationship between the variables that SRD validates. Fig 2 demonstrates the workflow of our method.

### Problem formulation

Multi-objective optimization problems can be interpreted as the minimization of a set of objective functions where each *f*_*k*_(**z**_*i*_), *k* = 1…*n* objective function measures the appropriateness of the *i*th solution represented by **z**_*i*_ vector of variables, **z**_*i*_ = [*z*_*i*1_, *z*_*i*2_, …*z*_*in*_], *i* = 1, …, *N*

$$\begin{array}{c} min\{f_{1}\left(z\right),f_{2}\left(z\right),\ldots ,f_{n}\left(z\right)\},k=1\ldots n \end{array}$$ (1)

The classical approach to solve the optimization problem is to find the set of Pareto optimal solutions. One solution dominates [31] (**z**_*i*_≺_*P*_ **z**_*j*_) another, if it is not worse than the other in any objectives, and is strictly better in at least one objective.

$$\begin{array}{c} \forall_{k}f_{k}\left(z_{i}\right)\leq f_{k}\left(z_{j}\right)\wedge \exists k|f_{k}\left(z_{i}\right)<f_{k}\left(z_{j}\right),\;k=1,\ldots ,n \end{array}$$ (2)

where *k* denotes the running index of the objectives, **z**_*i*_ and **z**_*j*_ are considered as the realizations of the variable **z**, while *i* and *j* denote the (indices of) solutions so that *i* = 1, …, *N*;*j* = 1, …*N*;*i* ≠ *j*.

The *f*_*k*_(**z**), *k* = 1…*n* objectives can conflict with each other, or can be redundant so the optimization problem becomes more difficult to solve. If the optimization consists of only a handful (*n* < 4) of objectives, then it is called multi-objective optimization. Including any additional objectives yields a many-objective optimization (MaOO) problem that retains the *curse of dimensionality*.

**Fig 3:**
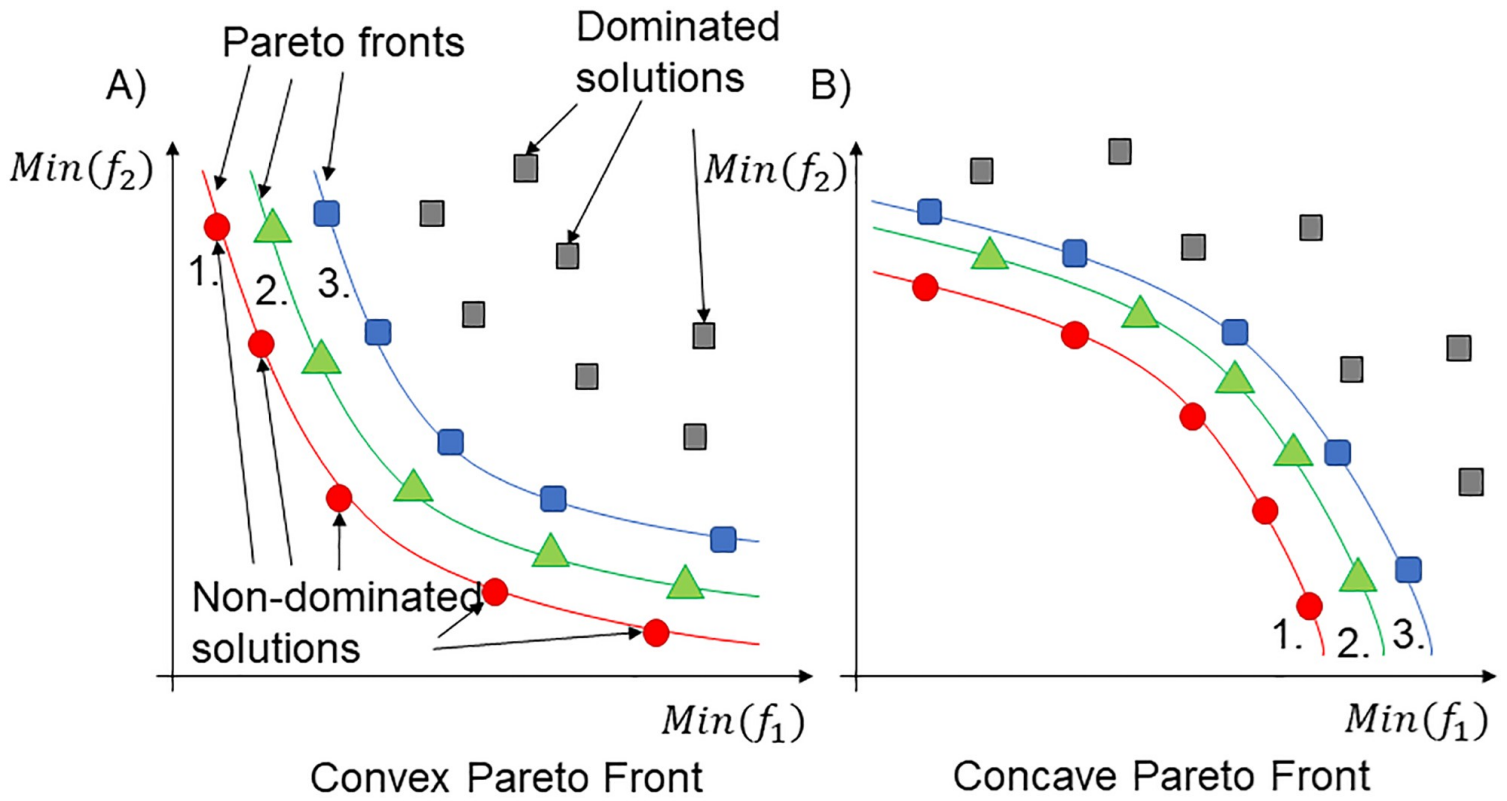
Pareto front representation. Pareto fronts can be convex (A), concave (B), or the mixture of both. Additionally, the set of solutions can have multiple Pareto fronts, which can be identified by ignoring previously determined non-dominated solutions and repeating the search for Pareto fronts. Consider an onion; peel off the first layer to see the second, which will then become the outermost layer. The Pareto front can also be clustered.[34, 35].

Pareto fronts can take up convex and concave forms or the mixtures of both. The border proposes different trade-offs. A concave Pareto front consists of strong trade-offs, while a convex front has relaxed ones [32]. There can be more than one Pareto front; if the first set of non-dominated solutions is discarded from the selection procedure, then the second front set emerges, the third, and so on until the number of solutions to form another Pareto front is insufficient. Multi-layered Pareto optimality [33] is analogous to an onion. When one removes a layer, the next set of dominated solutions appears; this procedure continues until the core is reached. The two extrema (the concave and convex cases) of Pareto fronts are illustrated in Fig 3. The non-dominated solutions are denoted by red circles, while the members of the second and third Pareto fronts are marked by green triangles and blue squares, respectively. The members of such Pareto fronts can be further clustered for post-Pareto optimality analysis [34, 35]. The MATLAB implementation of the Pareto front is provided by Yi Cao [36].

When the number of objectives increase drastically, the difficulties of MaOO problems [37] are enlarged because of:

- the deterioration of the performance of Pareto dominance-based search algorithms,
- the escalation in the number of solutions required to estimate the entire Pareto front,
- lack of visualization.

The collective name for the aforementioned difficulties is the *curse of dimensionality*. If the problem concerns dealing with several hundred objectives, the ranks are subjected to inflation, as more and more solutions obtain the same rank and hundreds will be firsts, seconds, *etc.* As a result, the number of non-dominated solutions expands exponentially, which causes the first two difficulties. The reason for the lack of visualization is the high dimensionality. One way to measure the curse of dimensionality is to apply Relative Entropy to the rankings of the objectives. As the number of objectives increases, the set of optimal solutions expands with additional ones [38].

The Pareto front requires consistent, aggregated objectives to handle the dimensionality. Without these conditions, the number of Pareto optimal solutions increases, and the front becomes saturated with each new objective resulting in a loss of meaningful information. Another important aspect of the problem is minimizing the number of redundant and conflicting objectives. The saturation of the front can also be measured with relative entropy [39] that is defined in subsection “The entropy and hypervolume-based evaluation of the ranking capacity”.

To study the problem, let us represent *x*_*i*, *k*_ = *f*_*k*_(**z**_*i*_) the value of the *k*th objective function of the *i*th solution. With this notation the *i*th solution can be realized by a vector that incorporates the evaluation of the *n* number of objectives:

$$\begin{array}{c} x_{i}=\left[x_{i1},x_{i2},\cdots x_{in}\right],i=1,\;\cdots ,\;N,\;x_{i}\in X \end{array}$$ (3)

where *i* is the running index of the solutions, **x**_*i*_ represents the *i*th solution out of *N*.

The *N* number of realized solutions and *n* amount of objectives are contained in matrix **X**_*N*×*n*_:

$$\begin{array}{c} X_{N\times n}=[\begin{array}{cccc} x_{11} & x_{12} & \cdots & x_{1n}\\ x_{21} & x_{22} & \cdots & x_{2n}\\ \vdots & \vdots & \ddots & \vdots \\ x_{N1} & x_{N2} & \cdots & x_{Nn} \end{array}] \end{array}$$ (4)

where the *x*_*ik*_ element of the matrix denotes a short hand notation for the *i*th solution’s value according to the *k*th objective.

The following subsection formalizes the developed non-negative matrix factorization-based solution developed to handle the problem of the *curse of dimensionality* with the key idea that the internal structure of the multi-objective decision problem can be extracted by non-negative matrix factorization of **X**_*N*×*n*_.

### Non-negative matrix factorization-based *many*-objective optimization method

The *curse of dimensionality* blocks the use of the Pareto front, yet the goal remains the same: select the best from a set of *N* solutions with all *n* number of objectives considered. Our main idea is to solve the problem with a general algorithm that works on any MaOO problem with a meaningful dataset, namely the sparse NMF. Dimensionality reduction provides a low-rank approximation of the original matrix, which consists of only a few aggregated objectives.

Our main focus is to reduce the number of objectives from *n* to *p*, where *p* < < *n*, but retain as much meaningful information as possible. The featured methods project the data to a lower-dimensional space:

$$\begin{array}{c} w_{i}=g\left(x_{i}\right) \end{array}$$ (5)

where **w**_*i*_ = [*w*_*i*1_, *w*_*i*2_…*w*_*ip*_] is lower dimensional projection of the realization of solution **x**_*i*_ by function *g*() to *p* dimensions. The reduced solutions are complied into a matrix denoted by **W**_*N*×*p*_. The reconstruction error measures the quality of the lower-dimensional approximation as the reduced set of objectives must retain the information content of the original set as much as possible. Thus our goal is to minimize the reconstruction error:

$$\begin{array}{c} \text{min}||X-\hat{X}{\left|\right|}_{2}^{2} \end{array}$$ (6)

where $\hat{X}$ denotes the reconstructed matrix from **w**_*i*_ low-rank approximation.

Most dimensionality reduction methods are built to reconstruct the original matrix from the projected space, aiming to represent the original matrix as accurately as possible. Therefore, the optimization problem seeks to minimize the difference between the original and reconstructed matrix.

**Fig 4:**
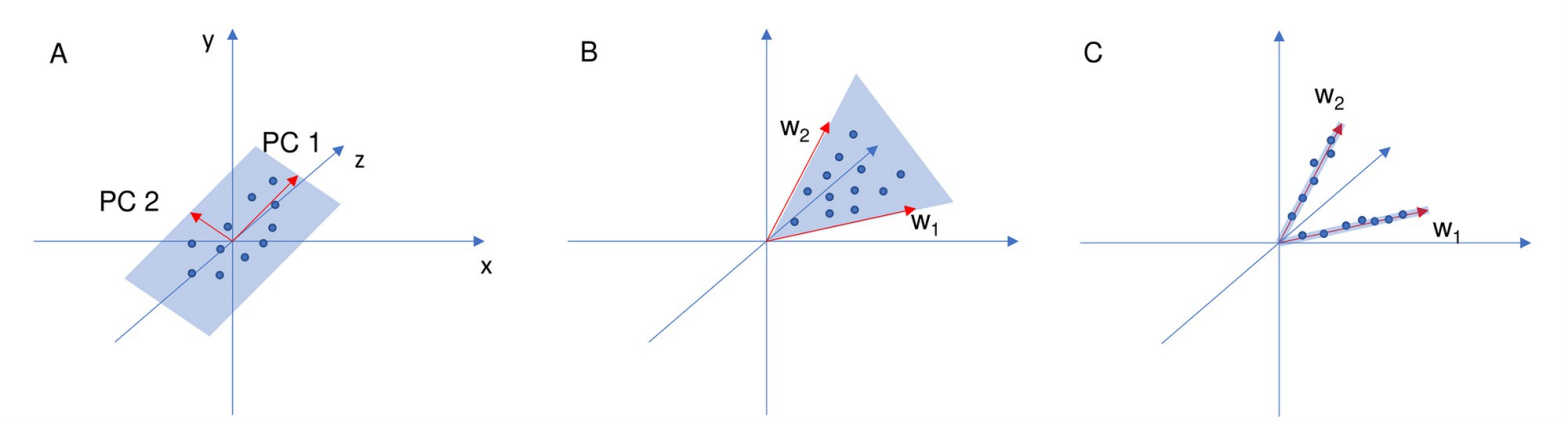
Graphical illustration of PCA(A), NMF(B), and sparse NMF(C). The matrix factorizations are reduced from three to two dimensions. PCA covers the solutions with a hyperplane, NMF constraints them between two vectors, and sparse NMF forces the solutions to be in the vicinity to the vectors.

In the present study, three dimensionality reduction techniques are applied, namely PCA, non-negative matrix factorization (NMF), and sparse NMF. The essential difference between them is depicted in Fig 4. The PCA (A) generates a hyperplane that covers all solutions, while NMF (B) takes two vectors and forces the solutions in between. Notice that PCA is capable of processing negative input and that the NMF and sparse NMF only utilize the first (positive) quarter of the Cartesian coordinate system. Sparse NMF (C) enforces sparsity and forces the solutions on the determined main vectors (or in their close vicinity). Changing the representations from A to C gradually generates fewer (and fewer) solutions, ensuring the sparse nature among the virtually endless number of solutions.

By reducing the number of objectives *n* to *p*, the MaOO problem devolves into a multi-objective optimization problem that can be solved by determining the Pareto front as dimensionality reduction decreases the effect of the *curse of dimensionality* sufficiently enough to be eligible for multi-objective optimization.

#### Principal component analysis

Principal component analysis (PCA) [2] is a classic dimensionality reduction technique. It is frequently used in methods for solving decision problems, such as in the PROMETHEE [40] method. PCA maximizes the amount of variance captured by the components sequentially. The principal components (PCs) are usually determined by calculating the eigenvalues of the normalized matrix. A hyperplane is created that spans over the solutions. The first component explains most of the variance and the following PCs retain less and less information. PCs with negligible variance percentages are discarded to reduce dimensionality, resulting in noise filtering and loss of information. Following singular value decomposition [41], the PCs can be determined by solving the following optimization problem:

$$\begin{array}{c} \underset{\left(W,H\right)}{\text{min}}||X-{WH||}_{2}^{2}\;,\;\;\text{subject}\;\text{to}\;\;W^{T}W=H^{T}H=I \end{array}$$ (7)

In terms of the PCA, **W** is often referred to as the score matrix, and is the projected data, while **H** describes the loadings (coefficients) of the matrix. As the requirement is that the product of the score, and the correlation matrices with themselves is to be one (**I** denotes identity matrix), both positive and negative numbers are allowed. The constraints ensures orthogonality, such that the aggregated objectives are independent of each other.

PCA reduced the number of objectives so that the effects of the *curse of dimensionality* are no longer a problem. However, the PCs incorporate negatively correlating objectives. The solutions of the Pareto front are both optimized in the negatively and positively correlating objectives (ranking those first who are the best in both a negative and positive objective). In contrast, the preferable outcome of a dimension reduction method would be only to optimize the positively correlating objectives. The novelty of this work is that non-negative matrix factorization is applied as its lower-dimensional representations are only composed of positively correlating objectives whereas PCA may provide negatively correlation objectives in the principal components.

#### Non-negative matrix factorization

Non-negative matrix factorization (NMF) [6] establishes a low-rank approximation to the input matrix in a low-dimensional space. To do so, matrix **X**_*N*×*n*_ is decomposed into **X** ≈ **W** **H**, where **W** denotes an *N* × *p* (score) matrix, **H** represents a *p* × *n* (loading) matrix. The number of reduced components *p* is significantly less than *n* (*p* ≪ *n*). **W** comprises the solution vectors (the rows of input matrix **X**), and **H** stands for the coefficient matrix comprising the objective vectors (the columns of input matrix **X**). For the elements of **W** and **H**, a non-negativity constraint is introduced, which allows positively correlating objectives only and creates non-orthogonal basis vectors. The constraint can also be perceived as vectors the solutions are placed between (such as in Fig 4). The optimization problem is mathematically formulated as follows:

$$\begin{array}{c} \underset{\left(W,H\right)}{\text{min}}||X-{WH||}_{2}^{2}\;,\;\;\text{subject}\;\text{to}\;\;W\geq 0,\;H\geq 0 \end{array}$$ (8)

where **X** denotes an *N* × *n* matrix, **W** represents an *N* × *p* matrix, **H** stands for a *p* × *n* matrix and *p* ≪ *n*. Here, *p* describes the number of reduced dimensions.

**Fig 5:**
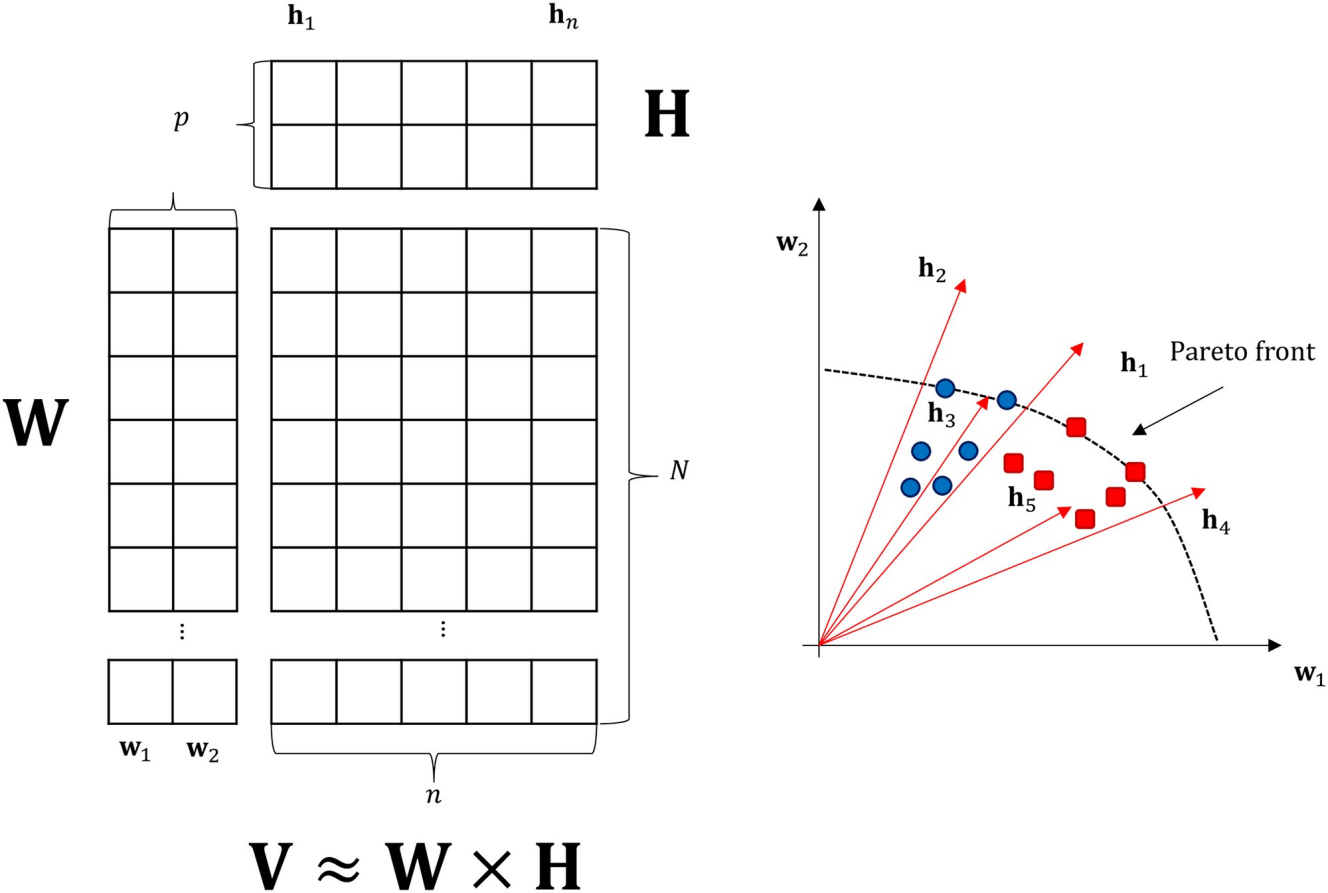
Illustration of non-negative matrix factorization in two dimensions. When the linear combination of **W**_*N*×*p*_ and **H**_*p*×*n*_ is considered, an approximation ($\hat{X}$) of **X**_*N*×*n*_ is obtained. Moreover, the technique has inherent clustering properties, with which the objectives are automatically clustered in **W** as are the solutions and the Pareto Front. **h**_*k*_ denotes the coefficients of objectives in relation to the components, which define the relationship between the objectives.

As presented in Fig 5, NMF is also capable of clustering; coefficients of the objectives in **H** determine how similar an objective is to a component. **W** returns the weights/scores of the solutions. The weights and coefficients matrices are multiplied to reconstruct the original matrix. The clustering happens naturally when the matrix is decomposed. The Pareto front placed on the reduced data points is also clustered.

To efficiently calculate the **W** and **H** matrices, the approximation is done through two equations that are iteratively solved.

$$\begin{array}{c} H^{z}=H^{\left(z-1\right)}\frac{{\left(W^{\left(z-1\right)}\right)}^{T}X}{{\left(W^{z-1}\right)}^{T}W^{z-1}H^{z-1}} \end{array}$$ (9)

$$\begin{array}{c} W^{z}=W^{\left(z-1\right)}\frac{X{\left(H^{\left(z-1\right)}\right)}^{T}}{\left(W^{z-1}\right)H^{z-1}{\left(H^{z-1}\right)}^{T}} \end{array}$$ (10)

where *z* denotes the index of iteration, and ^*T*^ stands for transposing a matrix.

The starting matrices (**W**^(0)^, **H**^(0)^) are filled with random numbers in the worst-case scenario and the equations approximate **X** iteratively by using the newest iterate of the other lower rank matrix.

By reducing the *n* number of objectives of the matrix **X** to *p*, the *curse of dimensionality* disappears. The Pareto front is applied by substituting the **W**_*N*×*p*_ into Eq 2, obtaining the ranks of the solutions *r*^*w*^, which can be sorted to select the best solutions. The reduced number of dimensions (new aggregated objectives) causes the Pareto fronts to be no longer crowded by the non-dominated solutions. The efficiency of the Pareto dominance-based search is reassured, and the visualization problems are solved. Only a positive correlation between the objectives is allowed due to the non-negativity constraint. Therefore, the optimization only considers the Pareto front of positively correlating objectives. The two or three dimensions determine the components that group different objectives.

The choice of input matrix **X** for the projection can be arbitrary, depending on the purpose of its application: the normalized form of the matrix provides information on the relationship between the solutions, while its ranked form describes the relationship between the ranked objectives.

#### Sparse non-negative matrix factorization

Our motivation is to remove irrelevant variables to improve the clarity of the analysis. Thus, NMF is further constrained with sparsity, which aims to represent the original dataset with the least amount of objectives. In other words, the aim is to reduce the number of objectives so that Pareto fronts can be applied and be coherent. Sparsity measures how many zero values are located in a matrix. In sparse NMF, the coefficients and weights are constrained to contain a number of zeroes based on regularization parameters. In a sparse matrix, the non-zero values can effectively represent the solutions, a consequence of which is the emphasis of non-dominated objectives. The constraining vectors that define non-negativity require the solutions to be placed on them, or in their vicinity. The required accuracy determines sparsity. By enforcing a certain sparsity, only the dominant objectives are defined in each constraint vector. The effect of sparsity is depicted in part C of Fig 4.

The usage of sparse non-negative matrix factorization (sparse NMF) [3, 4] is preferable to acquire a sparsity level. The method may enforce sparsity on both **W** and **H**:

$$\begin{array}{c} \begin{array}{c} \underset{W,H}{\text{min}}||X-{WH||}_{2}^{2}+{\alpha ||W||}_{2}^{2}+\beta \sum_{k=1}^{n}\left|\right|h_{k}{\left|\right|}_{2}^{2}\;,\;\\ \;\text{subject}\;\text{to}\;\;W\geq 0,\;H\geq 0 \end{array} \end{array}$$ (11)

where *α* denotes the regularization parameter of sparsity in **W**, while *β* enforces a sparseness constraint for each **h**_*k*_, *k* = 1, …, *n* columns of the **H** coefficient matrix to ensure sparseness of the model.

Several iterations need to be performed to calibrate both core and sparse NMF, the update function is based on the alternating least squares. For the sparse NMF, this function is similar to a variant of the Tikhonov regularization, namely the (*L*_2_) two-norm calibration or maintenance without reference solutions [42].

In summary, the Pareto fronts cannot handle high-dimensional input matrices as they become oversaturated. Therefore, the number of objectives must be reduced similarly to what occurs during PROMETHEE combined with PCA [40]. In this case, PCA may contain negatively correlating objectives in (any) single PC. The NMF tackles this problem by constraining the reduced dimension into the non-negative objective space. The consistency and clarity of the analysis can further be improved by applying sparsity to NMF, which removes redundant objectives.

### Tools for the validation of the NMF-based many-objective optimization

Although the NMF and sparse NMF techniques are widely-used and general methods, the implications of the novel use and the uncovered relationship between the objectives must be validated. The sum of ranking differences technique is applied for validation as it excels at measuring the similarity of the objectives to an ideal one. Moreover, the established Pareto front must be qualified to ensure the applicability of NMF. As the selection of the set of non-dominated solution operates on rankings, the over saturation of the ranking (the layers) and the handling of the *curse of dimensionality* can be measured with Relative Entropy and Hypervolume.

#### Validation by the sum of ranking differences method

The sum of ranking differences [7–9] is a novel statistical method that compares the objectives to an ideal or a chosen objective if the former is unknown. In other words, the main steps include the selection of the ideal reference objective. Subsequently, the objectives are ranked, and the City Block (Manhattan) distances are calculated from the reference objectives. Though it is not mandatory, the input of SRD algorithm can be preprocessed.

The SRD can be formalized as:

$$\begin{array}{c} SRD_{k}=\sum_{i=1}^{N}|rank\left(x_{i,k}\right)-rank\left(\rho_{i}\right)|,\;k\in \;\left\{1,\ldots ,n\right\} \end{array}$$ (12)

where *SRD*_*k*_ defines the similarity of an objective to an ideal objective, *rank*(*x*_*i*, *k*_) stands for the rank of the *i*th solution according to the *k*th objective, while *rank*(*ρ*_*i*_) denotes the rank of the *i*th solution *ρ*_*i*_ in the ideal reference *ρ*.

This method is an easy-to-use measure; the smaller the sum, the closer the objective is to the ideal ranking. The SRD is not a simple distance metric like the Spearman’s footrule, because it contains partial ranks (ties) and two validation steps: i) a randomization test and ii) a leave-many-out cross-validation [9, 20].

The cosine similarity of the constraint vectors of NMF may measure correlation and is capable of determining the similarities of the rankings. As such, if some objectives only reversely correlate to the constraint, these can be removed from the analysis due to their disrupting effect on the matrix factorization. Yet, to validate the findings, another well-established method should be used. An interesting result of this work is that applying the NMF on a ranking matrix yields about the same order of objectives as the SRD, with maximum ranks of the solutions as an ideal reference. However, the SRD provides an interpretable ordering of the objectives, whereas NMF only presents them based on their similarity to the constraint vector.

Substituting the ideal ranking (if it is unavailable) by approximation is also viable; aggregating the solution by a preferred method yields a reference, *e.g.*, row average, median, maximum, minimum, *etc*. The steps comprising the SRD are graphically described in (S1 Appendix) along with TOPSIS. Solutions and objectives are interchangeable from the viewpoint of the SRD. The proximity of SRD values suggests the relationship between the points; the closer they are to one another, the more similar they are on the condition of the given distance to the ideal reference.

#### The entropy and hypervolume-based evaluation of the ranking capacity

To comprehend the oversaturation of the rankings or Pareto fronts, the amount of tied ranks should be measured. Relative entropy/Kullback–Leibler divergence (RE) [39] is a descriptive but straightforward measure:

$$\begin{array}{c} RE\left(D\right)=\frac{\sum_{r}\frac{D(r)}{N}\text{log}\frac{D(r)}{N}}{\text{log}\frac{1}{N}} \end{array}$$ (13)

where *D*(*r*) is number of solutions with rank *r* = 1, 2, …, *N*.

Rankings should contain as many different ranks as possible to distinguish the solutions. The number of tied ranks should be minimal, which can be measured with the help of relative entropy that describes the distribution of the ranking [*D*(*r*)]. The smaller the entropy is, the more similar the values are, reducing the overall efficiency of the ranking and hindering the selection process of optimal solutions.

**Fig 6:**
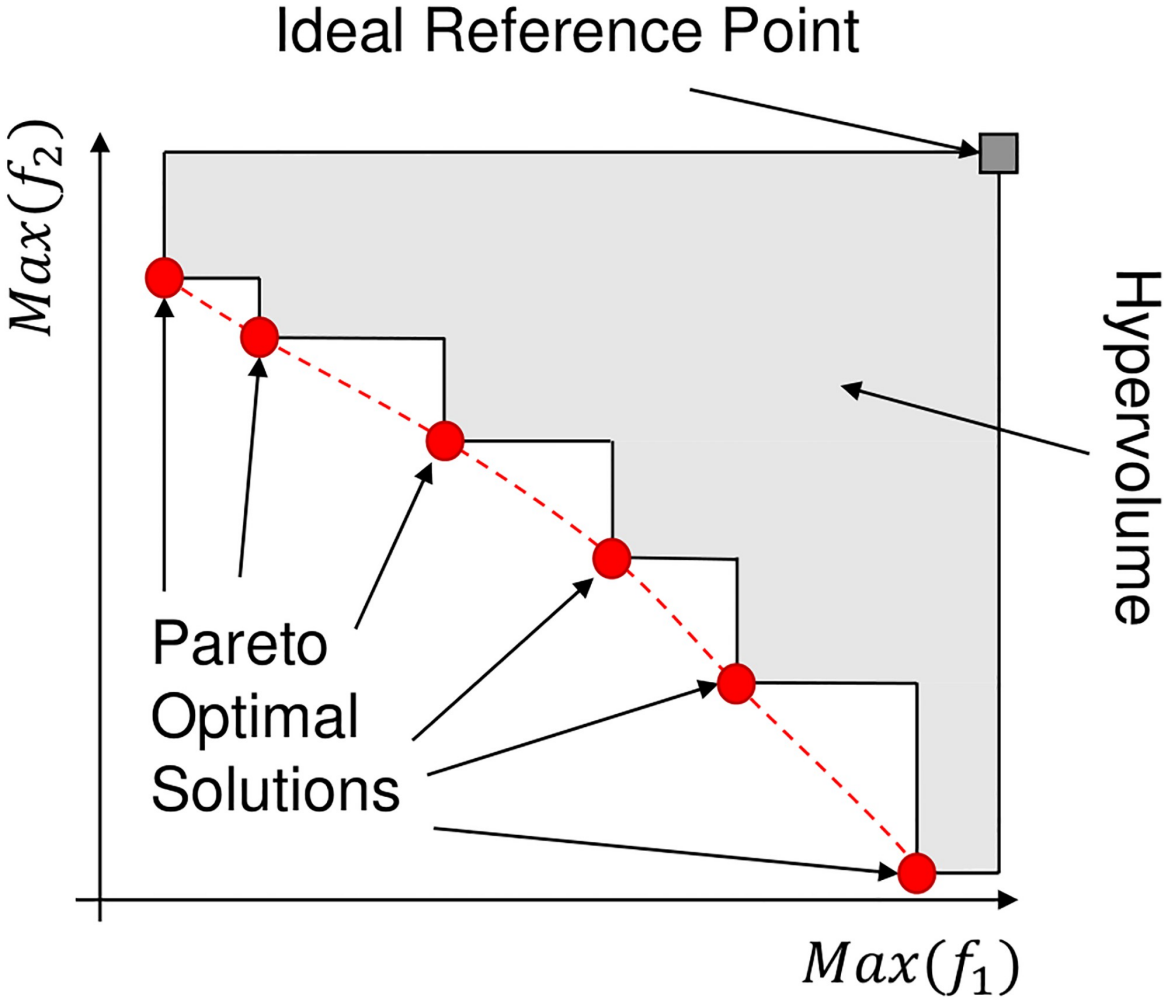
Hypervolume. The hypervolume indicator measures the volume between the Pareto front and the chosen/ideal reference. A Monte Carlo simulation approximates the Hypervolume measure due to its quickness. The red dashed line illustrates the Pareto front, while the grey area refers to the calculated hypervolume. If both the reference, and the Pareto front consists of maximum values, then the hypervolume is preferably close to zero. The illustration describes the calculation of hypervolume in 2D space.

The hypervolume [43] is used to describe the volume (area in 2D) between the front and an ideal reference to evaluate the convergence of the Pareto front. In this publication, the maximum values of the objectives are used as reference points. The area or hypervolume between the ideal point and the non-dominated set is to be minimized if the Pareto front is based on the maximum values, and *vice versa*. The algorithm provided by Yi Cao [44] estimates the hypervolume using a Monte Carlo [45] simulation which generated several evenly distributed points in the feature space to approximate the ratio of dominated points to total points, yielding a percentage as the performance measure that is proportional to the volume. Fig 6 presents the depiction of the volume between the Pareto front and the reference point.

The reason behind employing these measures is to evaluate the Pareto front. High hypervolume indicates an inaccurate Pareto front as considered a far cry from the ideal front. Low RE can be associated with over-saturated Pareto fronts that demonstrate the *curse of dimensionality*.

## Results

### CWTS Leiden Ranking 2020

We employ the CWTS Leiden Ranking 2020 database [10], which is based exclusively on bibliographic data from the Web of Science database. The database includes 1176 universities worldwide based on 46 objectives. The universities are selected from the Web of Science database based on the number of their indexed publications between 2015 and 2018. It is known that Web of Science, *ab ovo*, is biased towards the English-speaking scientific community, *e.g.*, the start of some rankings based on their data mainly includes institutes from English-speaking countries.

Two different methods are applied to preprocess the data. First, the min-max algorithm scales the objectives to the interval of zero and one, referred to as scaled input. The other method was ranking with tied ranks where the arithmetic means are designated to each solution (university) with equal footing (ranked input). Additionally, in the SRD analysis, the maximum value of each row (*i.e.*, the ranks of the university according to the objectives) is used as a reference, and no preprocessing was performed on the data.

The names of the objectives are shortened to the combination of a letter and a number. The letter indicates the type of the indicator, *e.g.*, scientific impact indicator, while the number describes the variable. For example, S2 is the abbreviation of the second of the scientific impact indicators, namely ‘the total number of citations of the publications of a university’. The list of abbreviations is provided in (S4 Appendix). To stimulate further research, the resultant MATLAB codes of the proposed non-negative matrix factorization-based ranking and visualization algorithms are publicly available on Zenodo.

### Ranking of the highest entropy pair of objectives

**Fig 7:**
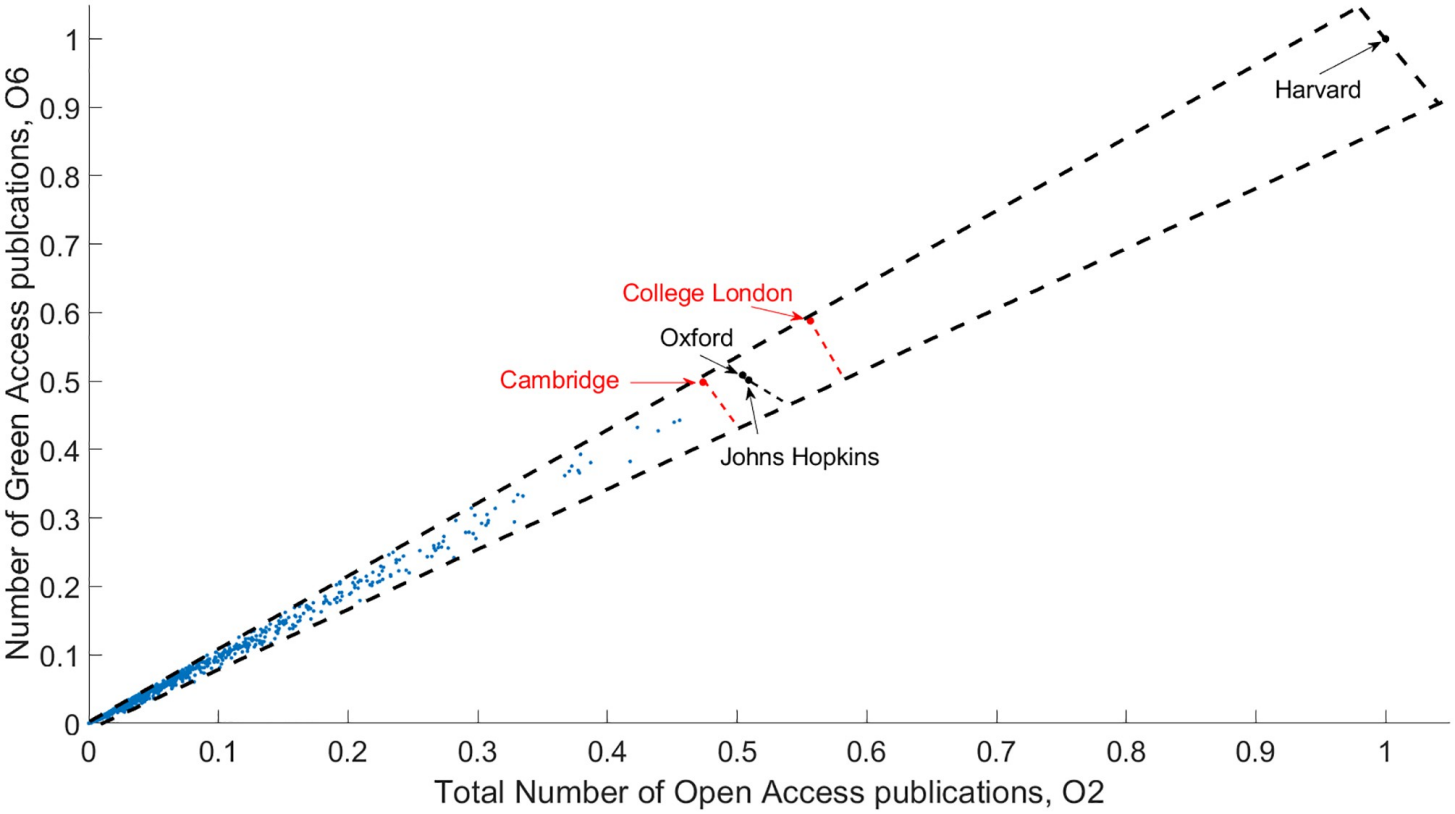
Pareto Front of the two objectives with the highest entropy. The Pareto fronts only consider those non-dominated solutions that are optimal from the viewpoints of the ‘total number of open-access publications’ (O2) and the ‘number of green access publications’ (O6). Coincidentally, the chosen objectives are one of the most correlating pairs. The other 44 objectives have no input to the ranking. Should another two dominant objectives taken into consideration, different non-dominated solutions would be obtained.

Concerning many-objective rankings, several questions may arise: How can ranking be fair? By being naïve and in the name of equity, considering all institutions firsts? Should all the positive and negative aspects of a university be considered? From the viewpoint of MaOO, if all solutions are optimal, it is not an optimization problem. However, if all objectives are incorporated to provide an agglomerated ranking, each solution might be the best from a specific viewpoint. In this case, though everyone is a first, a comprehensive ranking cannot be disclosed. In Fig 7 the problem is presented by selecting the combination of two objectives with the highest relative entropy (calculated according to Eq 13). The selected objectives can be considered the most dissimilar ranking in terms of distribution. This pair of objectives is defined as the total number of open-access publications (O2) and green open-access publications (O6). Green access publications are available to read without the journal. Without considering the other 44 objectives, the two yield a narrow Pareto front and a decent relative entropy value of (*RE*(*D*) = 0.7925).

When the number of publications increases, more and more scientists decide to self-archive their publications. As these publications are accessible to everyone, a publication may target a wider audience, not just those who subscribe to the relevant journals. These two objectives positively correlate with each other. If more researchers are familiar with the work of the particular author, the higher the chance of collaboration or citing/referencing is. Even though these objectives produce the most distinctive ranking, a contradiction is proposed as one of the most correlating objective pairs is chosen. The non-dominated solutions in decreasing order of optimality are as follows: Harvard University, University College London, University of Oxford, John Hopkins University (the latter two are on the same front), and the University of Cambridge. The Pareto fronts are visually separated.

The hardship of optimizing *many*-objective problems is that differentiating between the solutions is impossible, which is measurable with the relative entropy. On the other hand, when all the objectives are used as inputs, the ranking inflates, and the *curse of dimensionality* appears, resulting in all solutions being placed on the first Pareto front. In this case, the relative entropy is zero. The smaller it is, the more saturated the ranking and the fronts are. As a result, the following question remains: how is it possible to determine the best when all universities are on the front?

### The correlation structure of objectives and solutions

**Fig 8:**
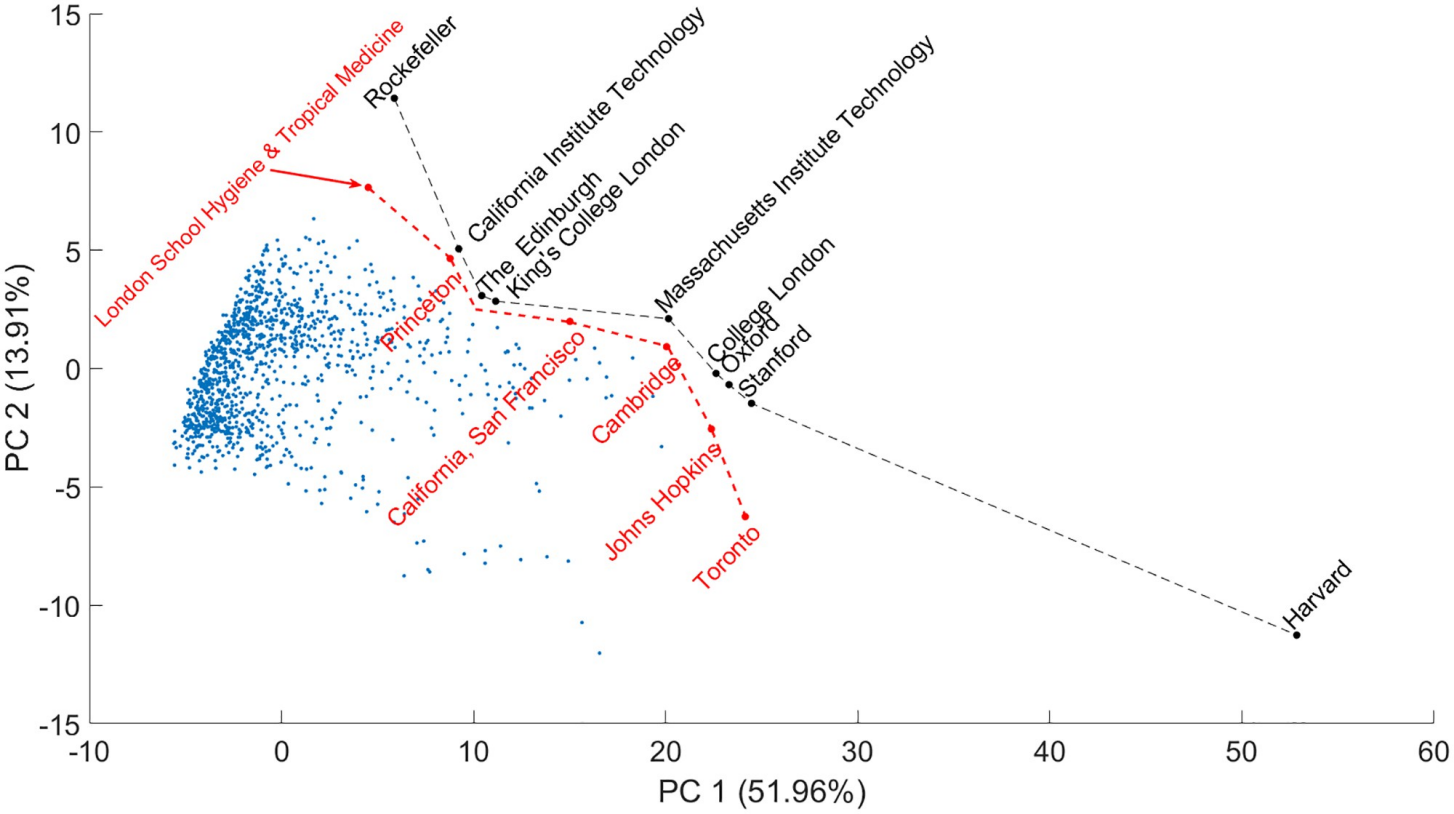
PCA-based Pareto front. In the first PC, objectives that focus on quantity are dominant, while the second PC promotes a high proportion of publications that perform well as scientific indicators, open-access as well as in terms of collaborations. Rockefeller University is a small private university but has an outstanding proportion of objective values compared to other, more robust universities. At the other end is Harvard University, where resources are available to create an immense amount of publications. Note that only universities from the Anglosphere are on the first two Pareto fronts.

The introduced dimensionality reduction-based techniques can solve the problem of the inflated ranks of many-objective ranking. The main idea is to reduce the number of dimensions while simultaneously retaining as much information on the original data as possible. Although the performance of NMF and sparse NMF is presented in this paper, the traditional PCA is also applied to provide a benchmark for the performance of dimensionality reduction, as well as present the correlation structure of the objectives. Only the first two principal components (PCs) were selected, which explains 65.87% of the total data variance. The Pareto fronts of the objectives are determined in this reduced objective space as presented in Fig 8. The relative entropy of the PCA-based ranking is *RE*(*D*) = 0.6072.

**Fig 9:**
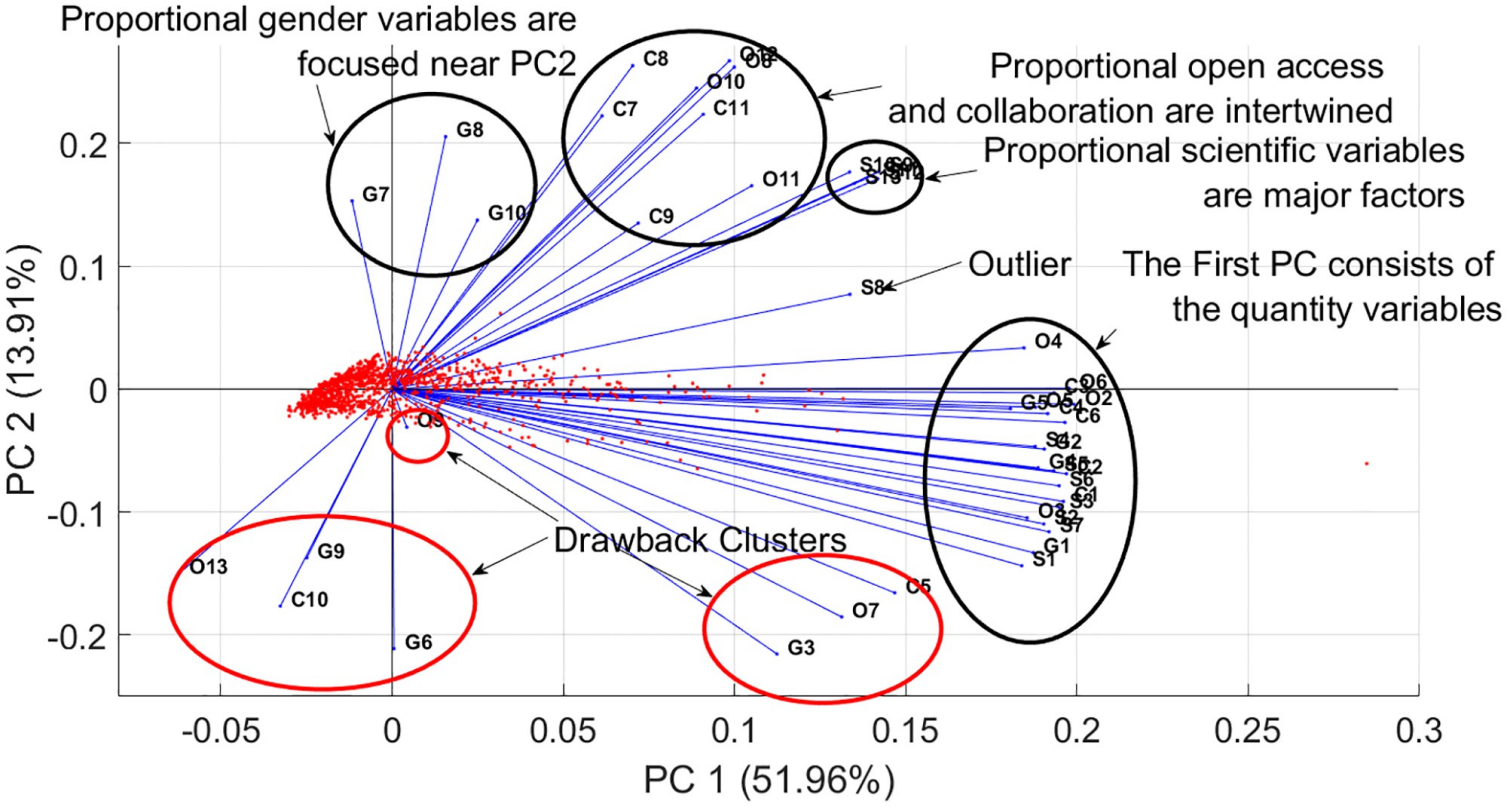
Relationship between objectives by PCA. The biplot depicts both scores and loadings, however, the scores are scaled differently in Fig 8. The axes split the objectives; the vertical axis distinguishes between the effects of the objectives that either impair or benefit the solutions, in this case, universities. In the fourth quarter, the objectives are quantity indicators, while in other quarters, the proportional indicators can be identified. Six clusters can be observed: three gender proportional indicators, the proportional variables of collaboration and open-access indicators, the proportional variables of scientific indicators, the quantity indicators, and the two drawback clusters. There is an outlier objective (S8). When a university improves their indicators in the drawback clusters, their performance decrease, moving the farther from the Pareto front.

Fig 9 depicts the correlation structure of the objectives on a biplot in two-dimensional space (solutions and objectives are displayed as points and vectors, respectively). The institutions that perform better in the first PC (51.96% explained variance) have produced many notable publications, reflecting the quantity aspect. In contrast, the second PC (13.91% explained variance) yields a high proportion of publications with a considerable scientific impact, open-access, and collaboration. Thus the second PC contains the proportional aspects of the ranking.

The loading vectors form six major clusters in Fig 9. The first two PCs (1–2) deal with the quantity and proportional indicators, respectively. The massive cluster on and in the vicinity of the horizontal axis contains the quantity variables of different indicators from which PC1 is mainly composed. PC2 summarizes the proportional components. It is positively influenced by G7 and G8, namely ‘the number of male and female authorships as a proportion of total number of authorships of a university’, respectively. Moreover, G10 can also be found in the vicinity, namely ‘the number of female authorships as a proportion of the number of the known male and female authorships’ of a university. The drawbacks exhibit negative gradients; these are the objectives that disadvantageously affect the rank of an institution. These form a major and a minor cluster; the major one incorporates the unknown gender (G6), the male-to-female ratio (G9), the proportion of unknown open-access publications (O13), and the proportion of short-distance (geographical) collaboration (C10) variables, while the members of the second minor cluster are the quantity equivalent of the latter three objectives from the first cluster: number of short geographical distance collaboration publications (C5), the number of publications whose open-access status is unknown (O13) and the number of authorships at a university for which the gender is unknown (G3). Short-distance collaboration decreases the rank (and, in this sense, the judgment/rating) of an institution; an idea provides a possible explanation for this as old as the New Testament: “Truly, I say to you, no prophet is acceptable in his hometown.” Luke 4:24. Unknown open-access publications cannot be appropriately cited, do not have a DOI number, and are generally low-level contributions to obscure ‘predatory’ journals. Should they be unknown to the other scientists, it is as if the publication never existed. A high quantity alone is insufficient to provide a better rank because the quality is equally as important.

After discussing the main driving clusters of the first two PCs, the variables in the positive quarter of the Cartesian coordinate system are left unexplained. These variables form two major clusters, and an outlier, namely S8, the ‘average number of citations of the publications of a university’. The first cluster is the proportional objectives of collaborations (C7-C11) and green open-access publications (O12), except the proportional short-distance collaboration indicator, which is almost inversely proportional to this cluster. By publishing free information, the university reaches a larger and more diverse audience. In other words, more ideas may be generated. Moreover, if a collaboration is initiated, the rank of the institution might benefit significantly from the new accredited publications. The second cluster consists of the proportion of the most cited publications of universities (S9-S13). The following statements might be trivial, but the cluster gathers descriptors, which lead to the proposition that the more negligible impact of the publications produced by an institution is, the poorer the performance. The proportion of scientific variables (S8-S13) shows that innovation is one of the central roles of academic institutions; the ranking is heavily influenced by publications with higher degrees of impact and the average number of publications. However, it should be emphasized that the point cloud cannot be so nicely interpreted as for loadings. Mirroring the blue dots (at the end of the lines) through the origin preserves the opposite signs for loadings. The optimal solutions are considered for both negatively and positively correlating objectives. Hence, it seems necessary to compress all points and all information into the first quarter of the Cartesian coordinate system. The random and reversely ranked objectives should be determined using SRD.

### Distinguishing the differences between rankings

In the present work, the SRD is the perfect tool to determine the relationship between different objectives, hence underpin, validate and interpret the results of the NMF-based solution. The application of the SRD provides a list of objectives that are ranked closely, randomly, or reversely to the ideal rank.

**Fig 10:**
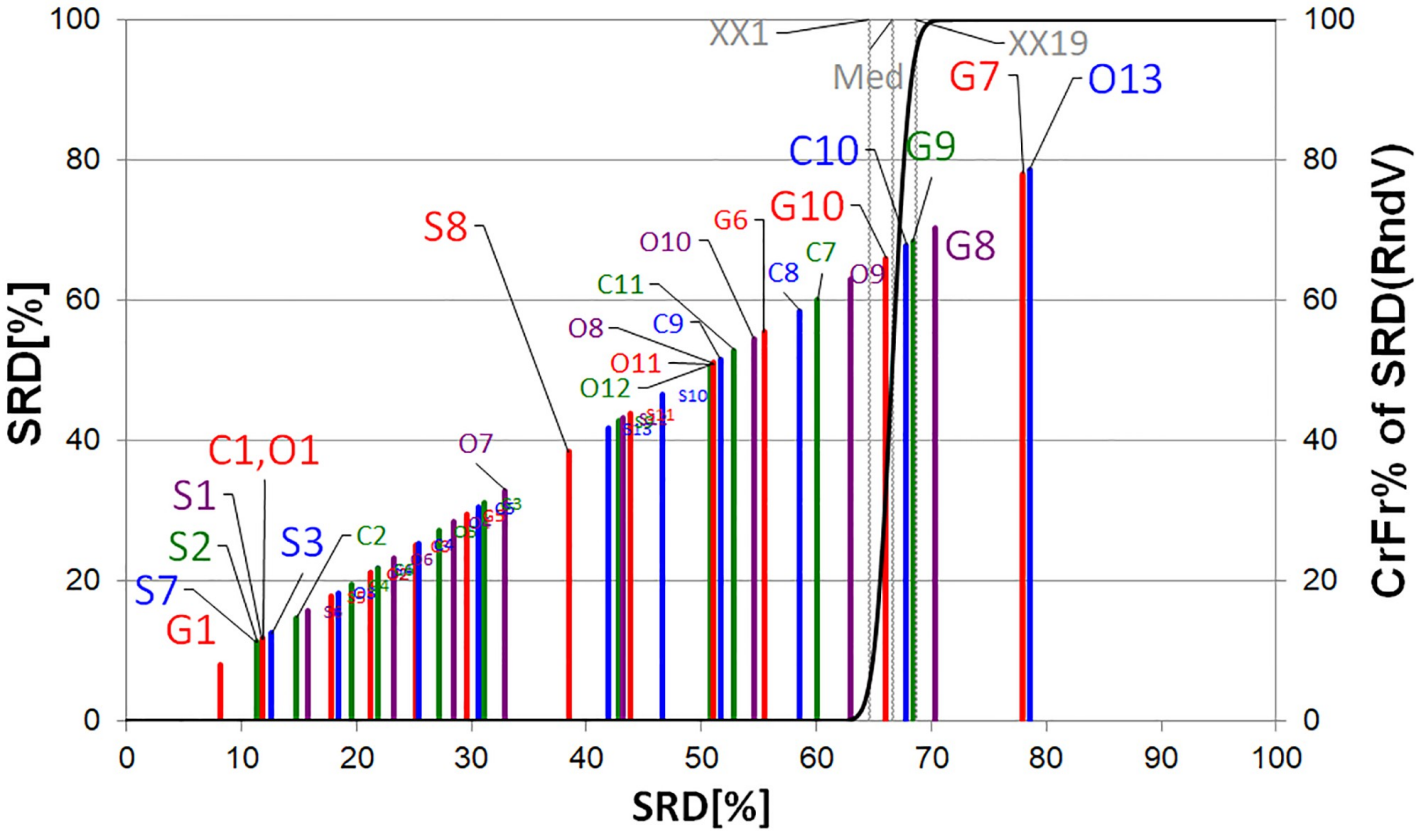
Representation of the objectives by SRD. The SRD range between 0 and 8 is empty, *i.e.*, the total number of authorships (G1) is close to the ideal reference. The range between the lines XX1 (5%) and XX19 (95%) shows the random ranking. Objectives to the right-hand side of the line of XX19 are ranked in reverse order (G7-G9, O13). The solid black line on the second vertical axis indicates the cumulative random frequencies of SRD vectors (CrFr% of SRD(RndV)).

Fig 10 illustrates the results of the SRD analysis. The information is presented according to the position/grouping of lines rather than their length. The solid black line indicates a cumulative Gauss approximation (normal distribution) of the ranking, and its value is indicated on the second vertical axis, namely the cumulative random frequencies of SRD vectors (CrFr% of SRD (RndV)). The objectives, whose positions are close to the zero rank sum, are similar to the golden standard/ideal ranking. The objectives, whose lines intersect with the probability curve (between XX1 and XX19), cannot be distinguished from the random ranking. The objectives to the right-hand side of line XX19 tend to be ranked reversely to the ideal ranking.

In terms of positive ranking, the ‘wrong’ objectives are located on the right-hand side of the XX19 line (randomly or reversely ranked indices). Moreover, similarly, the ‘good’ objectives are on the left-hand side of the XX1 line. The randomly ranked objectives can be identified between the two lines. The first five of both ‘good’ and ‘bad’ objectives are provided in Table 1. Presumably, a university that publishes (S1) as well as receives more citations (S2) is considered better. The universities with more authorships (G1) may be closer to the ideal reference. More publications in the 50% (S7) most frequently cited and increasing the number of collaborations (C1) positively impact the ranking.

**Table 1:** SRD without preprocessing. The worst objectives, in terms of positive ranking, are proportional indicators: short-distance collaborations (C10); male (G7) and female (G8) authorships in proportion to the total number of authorships and female authorships (G9) in proportion to the authors who disclosed their gender.

Rank	Worst Obj.	SRD value	Best Obj.	SRD value
1	**O13**	78.57	**G1**	8.17
2	G7	78.00	S7	11.28
3	G8	70.30	S2	11.36
4	G9	68.37	S1	11.81
5	C10	67.82	C1	11.84

### NMF based CWTS Leiden Ranking 2020

As some objectives in the PCA components negatively correlate to the other positive objectives, the Pareto front is selected along both negatively and positively correlating objectives which invalidates the ranking.

**Fig 11:**
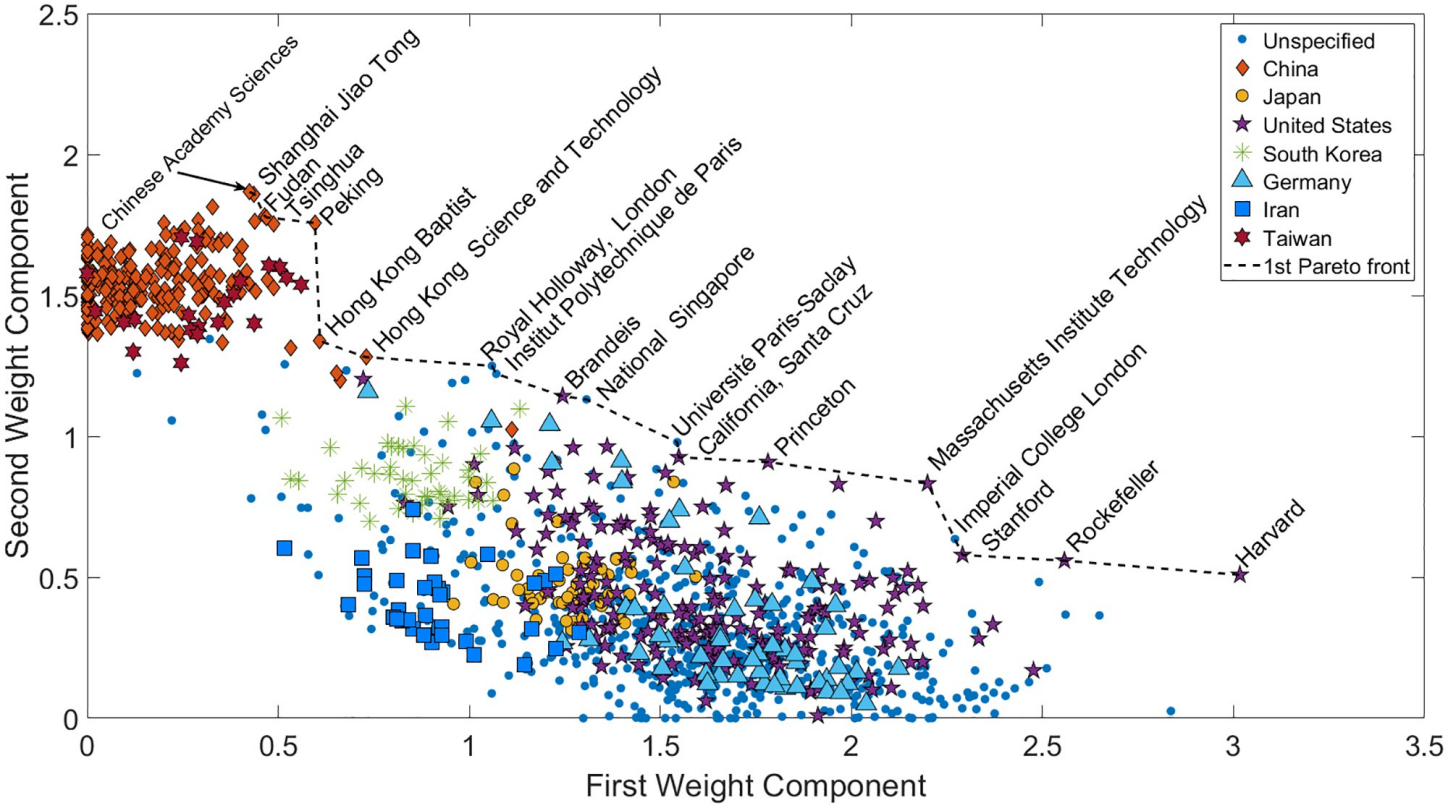
NMF-based Pareto front with scaled data as an input. Each indicator in the components is positively correlating with each other. Therefore there are no reverse objectives, only perpendicular ones. For this reason, the Pareto front is determined with the help of maximizing the objective functions. The NMF is applied to the scaled data before the Pareto front ranks the institutions. This method clusters the institutions into Eastern and Western cultures concerning education. The Chinese and Taiwanese universities are placed into a cluster on the left-hand side, while the European, American, and the remaining academies can be found on the right-hand side. South Korean and Iranian universities connect the two dominant scientific circles.

With Non-negative matrix factorization, both the original and ranked data are examined. In Fig 11, the upper section of the first Pareto front consists of Chinese institutions. By progressing along the line, British and American universities, as well as French and Singaporean ones, appear. The relative entropy of the result is *RE*(*D*) = 0.5131. It is also important to note that the components are maximized, as no negative correlation between the indicators can be established due to the non-negativity constraint.

The method inherently clusters the universities into institutions under Asian (mainly Chinese) and Western cultures. The brown diamonds and red stars’ cluster on the left-hand side contains Chinese and Taiwanese institutions. The South Korean universities appear first as a subcluster of the second bigger cluster by proceeding further right. Under that subcluster are the Iranian institutions close to the lower-ranked Pareto fronts. The universities above can be regarded as under the influence of both cultures found in Western and Eastern-style institutions. The institutions influenced by the West cover the remaining space as well as some subclusters are created by nations, *i.e.*, Japan. Germany and the United States cannot be distinguished properly, as their institutions are scattered throughout the West cluster. Some universities, such as Royal Holloway, and the University of London, are located much closer to the left-hand side cluster due to their willingness to enroll many students from diverse cultural backgrounds. Brandeis University focuses on liberal arts and offers various cultural studies. The Pareto fronts are clustered to provide a vivid multicultural ranking, where nationality is accounted for without distorting the ranking.

**Fig 12:**
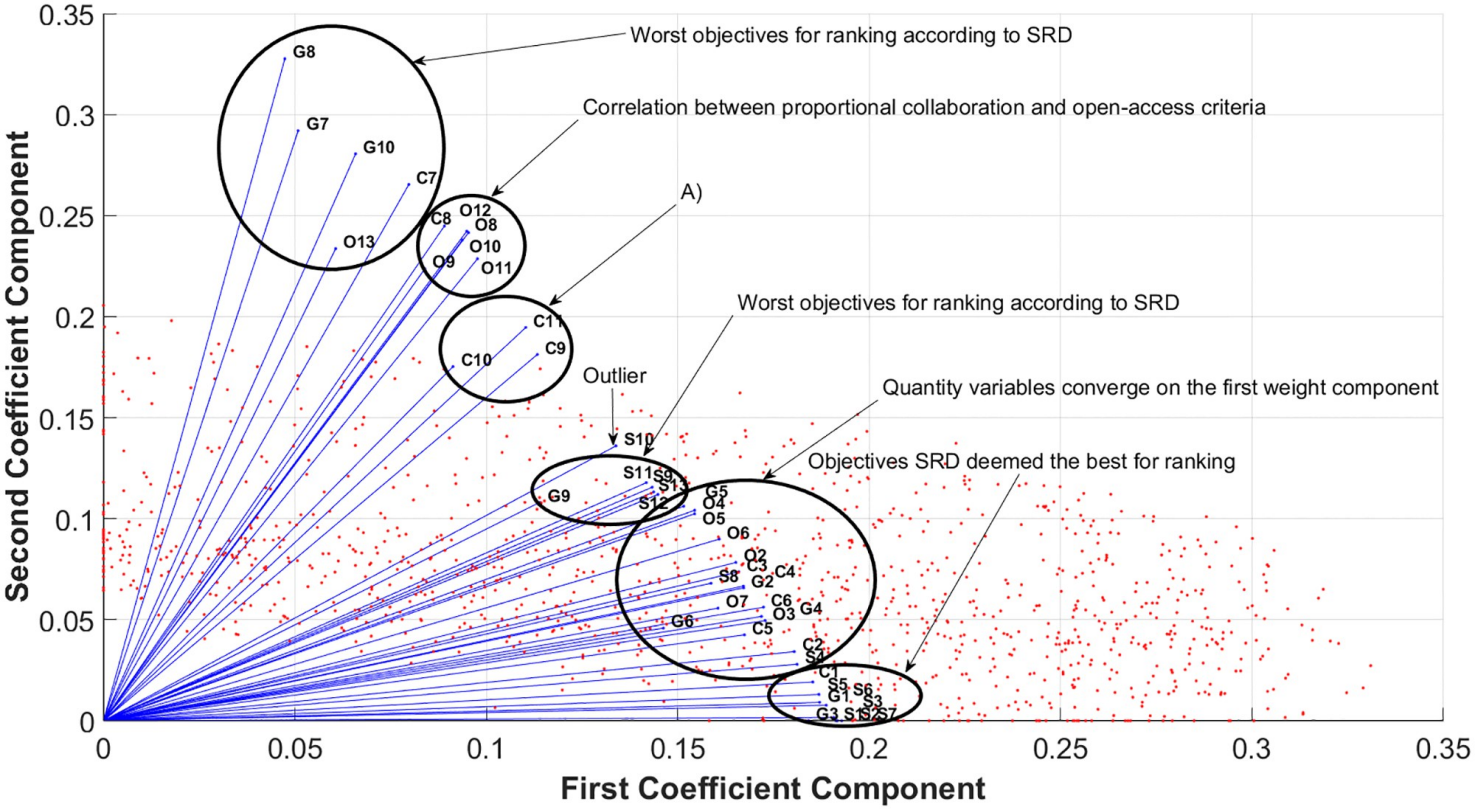
Coefficients of the ranking by NMF with ranked data as an input. The non-negativity constraint forces the objectives into the positive quarter of the Cartesian coordinate system. By providing ranked input to NMF, an insight was gained into the relationship between the objectives. The ones close to the axes are almost identical to the SRD result of best/worst objectives, but the randomly located ones are in between. Furthermore, clusters of indicators appear as well. Cluster A) describes the alignment of proportional collaboration variables (C9–10).

If the ranked matrix is chosen as the input of NMF, it provides information on the relationship between the ranks and ranked objectives of the universities. The biplot in Fig 12 describes these relationships. As ordering of the ranking in the NMF and the SRD with maximum reference provides the same ranking, we assume that the cosine similarity of the vectors, in this case, provide a measure similar to rank correlation. Both can be used for measuring rank similarities. In a clockwise direction, the clusters are the following: near the second component, as shown in the figure, objectives deemed the worst by SRD can be identified. From the next cluster, it can be deduced that the proportion of international collaboration (C8) is connected to open-access publications (O8–12). Various underlying reasons for this can be derived, *e.g.*, open-access publications may promote international collaborations, or some international funding opportunities encourage cooperation and open-access as performance indicators. The proportional collaboration indicators, namely, short-distance (C10), long-distance (C11), and industry (C9), are much more closely related than in PCA. Proportion of the top 1% cited (S10) is an outlier in this representation but similarly separates the proportional and quantitative indicators as in the PCA-based projection. The cluster under S10, consists of proportion of male authorships (G9), average number of citation (S9) and proportional scientific impact indicators S11–13. It suggests that the ranking of the male-to-female ratio weakly correlates to the proportional indicators of the 5–50% scientific impact and the average number of publications, which may be accredited to women being ill-represented in some highly-citing fields.

On the other hand, the next cluster (G5, O3–5) consists of female authorships and the quantity of hybrid, bronze, and green open-access publications. The remaining quantitative objectives are located somewhat close to each other, as is the case in PCA. These objectives form the intermediate components of the SRD values by connecting the outlier one with the best/worst objectives. The relationship between the objectives of the upper clusters is similar. NMF selected the same order of objectives that SRD chose for the first five in both directions. The best objectives in terms of ranking were placed in the vicinity of the first component. Given the non-negativity constraint, all objectives correlate positively.

**Fig 13:**
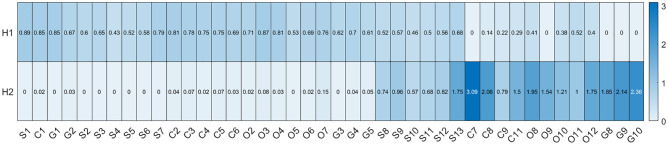
Heatmap of the coefficients in sparse NMF. Sparse NMF forces the coefficients of the objectives to take on the form of basis vectors, as well as sorts them into quantity (H1) and proportional (H2) objectives.

Sparsity on the scaled data was enforced to see what non-dominated factors represent the dataset. The ‘worst’ objectives were removed, and as a result, the components changed their identity to proportional and quantity components. Fig 13 depicts the values of the objectives in relation to the two basis vectors. The proportional scientific indicators are considered transitional between the basis vectors. Excellence in these objectives improves the rank of the institution in both components.

**Fig 14:**
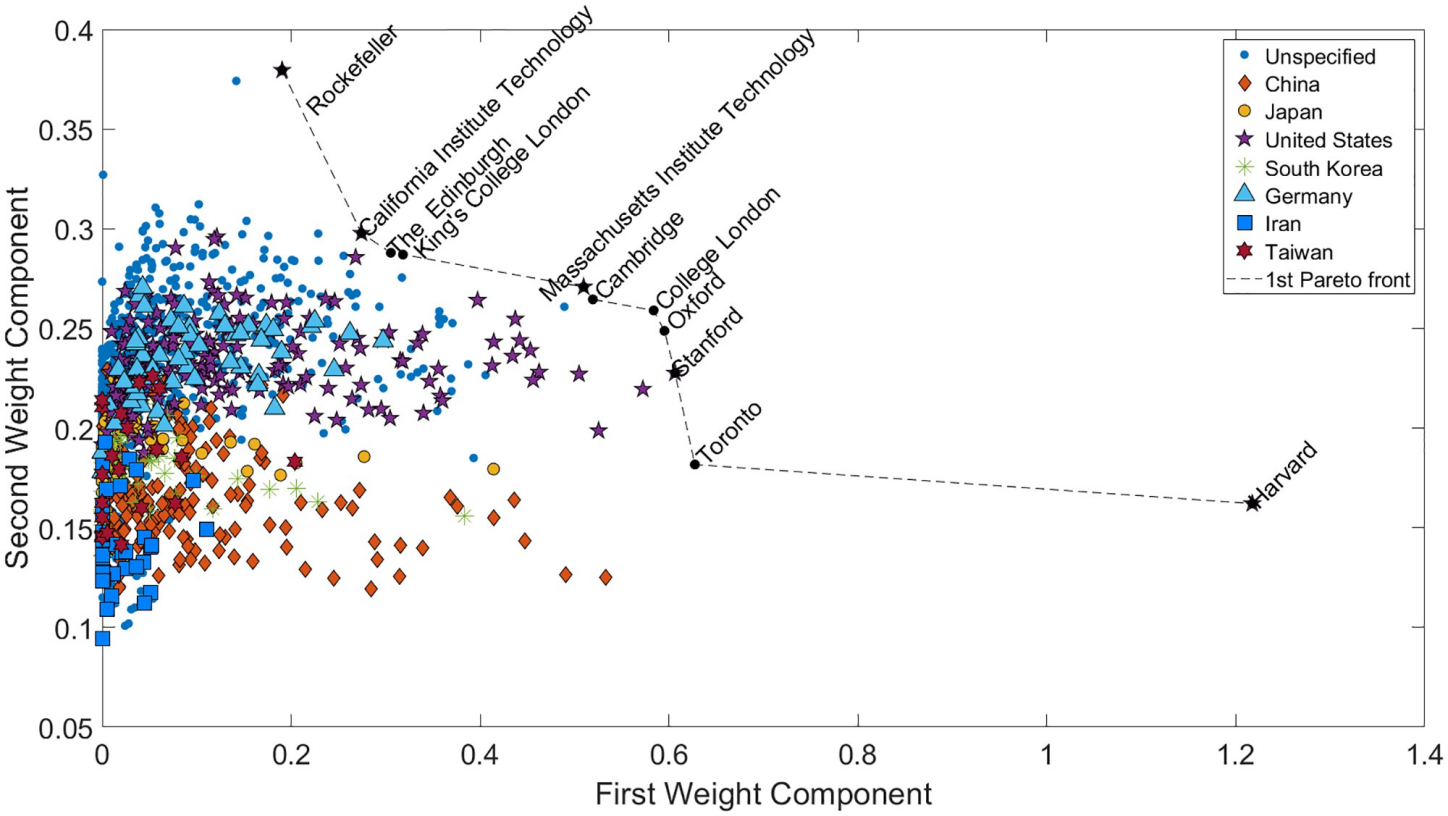
Pareto front of the sparse NMF. The *α* = 0.4 and *β* = 0.4 parameters are selected to incorporate a fair number of members to the Pareto front, and also have most of the objectives incorporated into a component.

Fig 14 draws the sparse NMF without the “worst” objectives. This caused the Asian information circle to gain its cluster. The objectives are constrained to one component, except for proportional scientific indicators (S8-S13). The two clusters are formed by the Asian (China, Taiwan, Korea, Japan) and Western institutions with a small intersection consisting of mixed institutions. The Western institutions are closer to the Pareto front. As a rule of thumb, the upper right an institution is placed, the better rank it gets; therefore, those who only perform exceptionally in the second component can be included in the second or third Pareto front. Generally, not only the number of the publications but their quality is considered important in terms of the performance of an institution. The best way to climb the ladder to the first Pareto front is to improve and excel at the proportional scientific indicators present in both components.

The front itself consists of institutions from the Anglosphere. It is important to note that even if these are exceptional institutions in terms of both components, the following Pareto fronts are filled with academies of Asian and other nationalities. A vital part of the sparse NMF method is the regularization parameters, selected to be 0.4 each. These balance the relative entropy and hypervolume to some extent; therefore, the determination may be possible with the help of the performance metrics, but further research is required for an exact iterative algorithm. As such, the number of the bests can be influenced, and the accuracy can be determined as to how many of the solutions should be placed on the Pareto front.

It is important to note that the benefits of the sparse NMF are the customization of the number of members of the Pareto front and a more precise representation of the components (as most objectives are present only as a part of one component).

### Discussion

Effects of the size and echo inherently distort the reliability of the results. In other words, more prominent universities have an advantage in terms of almost every objective indicator. Moreover, a once prestigious university will be highly regarded for many years to come, even if it can no longer maintain its outstanding performance. The rankings based on different objectives agree (and therefore are generally reliable) in terms of the leaders and drivers (the end of the ranking), while in the center region of the different rankings, the difference in relative ranking is often only visible through a magnifying glass [46, 47]. These findings are well-underpinned by the solution of the SRD analysis, in which the row maximum is used as the golden standard without preprocessing the data.

The indicator of ‘unknown gender’ is outstanding in the case of new universities inhibited by possibly smaller ethnicities. They may have a higher degree of uncertainty concerning the gender of their researchers, most likely determined by automated engines. It is a possibility that their universities are not ranked amongst the top universities due to their young history and the lack of publication records. Hence, the “unknown gender” indicator ranks in the reversed order.

The extended TOPSIS method [15] is capable of determining a suitable ranking even with a high number of objectives but does not provide comprehensive visualization and information on the relationship between the objectives. We have proved in Figs 11 and 12 that NMF can perform the latter two; moreover, the clustering of the institutions can be added to the toolbox of the analysis.

The only downside of the Weighted Top Candidate (WTC) selection method [29] is that the performance of the method is influenced heavily by the institutions as the recommendation system requires experts to propose new experts and that the method is sensitive to the structure of data. Compared to WTC, NMF can be fed with differently preprocessed inputs, which may result in a somewhat changed structure, *e.g.*, if rank transformation is applied to the data. The results return the relationship of the ranks, rather than the data, though the data is affected through a layer of abstraction. Besides, no ‘hierarchy’ exists in NMF; the clustered weights provide a Pareto front to be evaluated. It is important to note that WTC is not a method proposed to solve MaOO but rather an identification system for academics; therefore, comparing the two methods is to be handled accordingly.

Compared to the University Profiling Network (UPN) [30], NMF is a somewhat more transparent method as it does not require hidden layers. Autoencoders are prone to overfit the model, contrary to dimensionality reduction methods. The update function of NMF can be stopped by setting the maximum number of iterations or the maximum error; if Eq 8 reaches the preferable value, the algorithm stops. Although both methods are feature clustering ones, the main difference is that determining the relationships between objectives is much more suitable to NMF as it provides coefficients. At the same time, autoencoders are primarily closed and complex methods, making it difficult to pry into and visualize.

The trade-off ranking method [16] is sensitive as it utilizes the distance from ideal points as a means to determine the Pareto set. Regarding the difference between trade-off ranking and NMF, the former is not limited to non-negativity due to being a distance-based method. It focuses on the solutions that outperform others in each component. The latter reduces the number of objectives to the desired amount, clusters the Pareto fronts, and provides coefficients to explore the underlying data structure.

**Fig 15:**
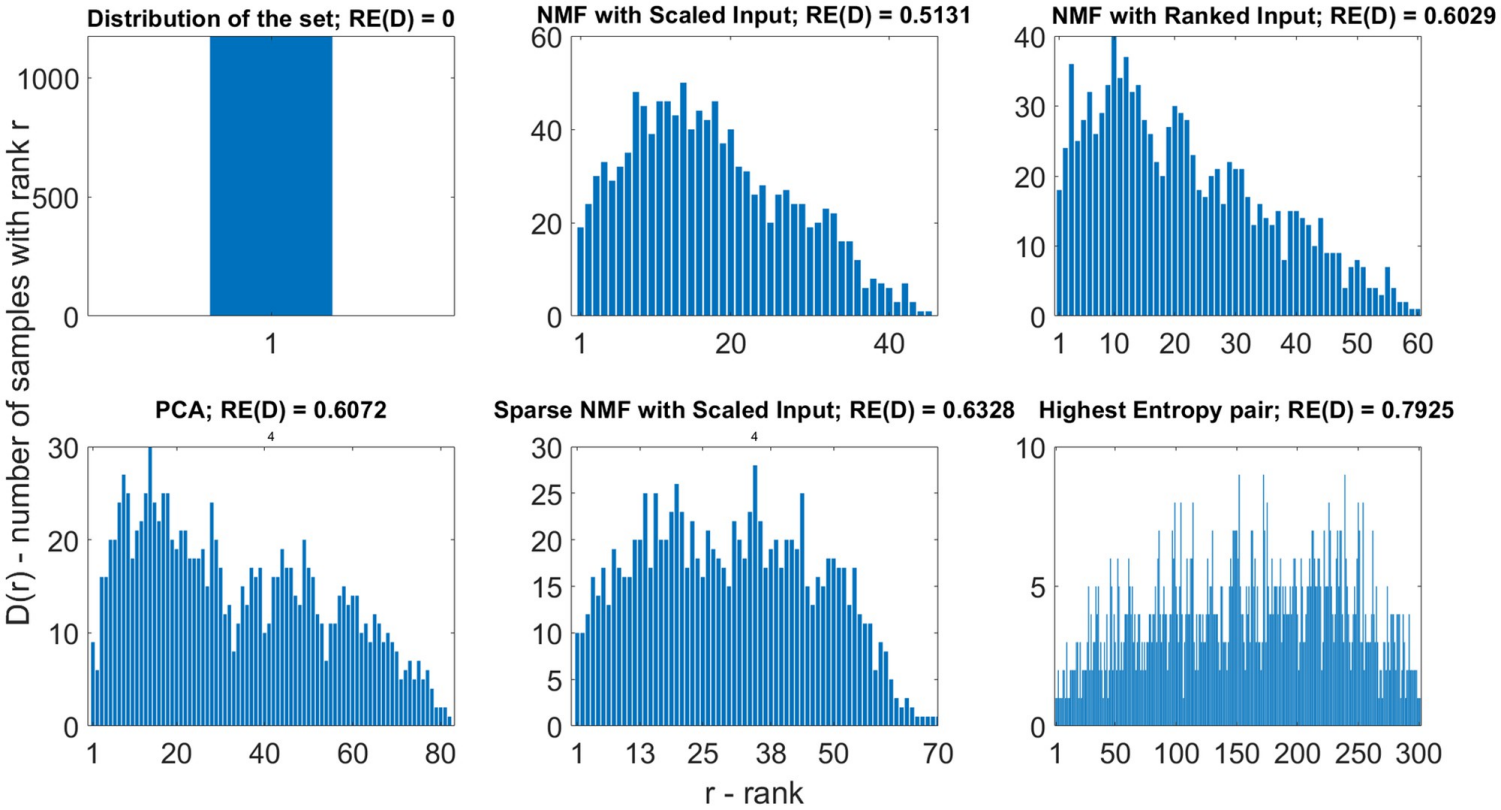
Relative entropies of the methods. The smaller the range of the y-axis (solutions with ranks), the bigger the range of the x-axis (ranks) is, and *vice versa*. Data with high REs have more ranks and fewer solutions with the same rank. The histograms represent the saturation of the Pareto fronts. From the viewpoint of Entropy, PCA and sparse NMF performs more efficiently than NMF. As the evaluation based on PCA consists of more ranks, the evaluation of the sparse NMF clarifies a more specific set of optimal solutions, which SRD validates. Furthermore, bimodal distributions can be observed (or suspected), except in the top left figure.

The RE of the dataset, the highest entropy objective-pairs, and each method seen are presented in Fig 15. The histograms represent the saturation of the Pareto fronts. If the RE is too small, the Pareto front is oversaturated, similarly to what is observed on the top left histogram, which provides the saturation of the dataset. In this case, the Pareto optimality cannot distinguish the solutions, and each one of the data points is placed on the Pareto front. The bottom-right histogram illustrates the highest objective pair depicted in Fig 7. This figure represents well-separated Pareto fronts with few members: one university for the first and second front, two for the third, and one for the fourth. The same number of solutions with ranks and universities on the respective Pareto front can be seen on the histogram. The other histograms show the saturation of the dimensionality reduction methods.

Table 2 illustrates the performance of the methods in the light of the relative entropy of the ranking and the hypervolume value of the first front (the distance to the reference point). The relative entropy measures ranking efficiency and hypervolume represents the closeness to the ideal solution. The ideal method would compress the objectives but retain high relative entropy and stay close to the ideal Pareto front with a low hypervolume value.

**Table 2:** Evaluation of performance. The ideal solution has a high relative entropy but minimal hypervolume, which is indicated by the arrows ((↑) represents that higher value is generally better, (↓) denotes that lower is preferable). The aim of dimensionality reduction is to compress the high number of objectives and retain as much information for ranking as possible. This results in a similar solution to the highest entropy pair, but takes all other objectives into consideration.

Method	Entropy ↑	Hypervolume ↓
Set	0	0.0178
Highest entropy pair	0.7924	0
PCA	0.6072	0.3602
Scaled NMF	0.5131	0.4016
Ranked NMF	0.6029	0
Sparse NMF	0.6328	0.2527

The Pareto front of the original dataset includes all universities; thus, the relative RE and hypervolume values are close to zero. The selection of the pair of objectives with the highest entropy provides an ideal solution as it efficiently determines the best university, *i.e.*, the first Pareto front is constructed from only one solution. In this case, the two chosen objectives are almost directly proportional. However, in this case, the other objectives are not considered, and by adding them, the problem of the *curse of dimensionality* returns. The NMF with the scaled input matrix is outperformed by all other dimensionality reduction-based techniques. Although PCA performs better in determining the ideal Pareto fronts as reflected by its lower hypervolume value, its drawback is that inversely correlated objectives are merged in its components, *i.e.*, with the cost of losing the interpretable and positively correlating objectives, PCA provides a more accurate approximation of the ideal Pareto front. The orthogonal PCA factors are not easy to interpret; similar variables may have different signs, SRD arranges them in 1D and shows the reverse (and random) characters of variables. The NMF groups the original variable in an easily perceivable manner. The clusters of objectives are no longer constructed of conflicting objectives. Sparse NMF provides a better ranking solution than PCA, as it clusters the similarly ranking objectives together (similar to general NMF). Sparsity can also be calibrated to control the number of solutions on the Pareto front, which comes in handy when the saturation of the Pareto front is to be controlled. Sparse NMF with ranked input has not been included because the ranks or the additive combination of ranks cannot be zero, and enforcing these invalid values reduces the efficiency and validity of the ranking. The relative entropy value of NMF with ranked input is not extraordinary. Yet, the hypervolume is zero, the smallest of the dimensionality reduction methods, providing a good trade-off between the results. Depending on whether the efficiency of the ranking or the closeness to the ideal solution is required, sparse NMF and NMF with ranked input is recommended, respectively. The time required was 0.5 seconds to approximate principal components, and the approximation of lower rank matrices (**W**_1176×2_ and **H**_2×46_) required 0.3267 seconds with an Intel Core i7–6700HQ 2.60GHz processor and 8 gigabyte RAM. NMF with ranked input was executed in 0.0389 seconds due to positive integers, and sparse NMF with removed objectives was performed in 0.3507 seconds.

The finding implies that the NMF can successfully solve MaOO problems. The non-negative objective space is reduced to the desired number of components that supports the generation of easily interpretable Pareto fronts. The proposed SRD-based filtering of the objectives is beneficial to remove non-informative and reversely-ranked objectives.

## Conclusion

This work focuses on ranking based on numerical values and their objective functions. Of course, subjectivity may influence the analysis in various ways; the most obvious is the selection of weights and redundant objectives, which deteriorates the analysis by showing the group of criteria more important than it is. The reason behind using the non-negative matrix factorization (NMF) is to reduce human subjectivity by automatically selecting matching objectives in one component, while reducing redundancy and removing subjective weighting of the objectives.

Any dataset may contain several objectives. The redundancy influences the weight of the objective groups. If the group comprises mostly irrelevant variables, the weighting will incorrectly shift toward that group. Without modification, the sum of ranking differences (SRD) and NMF can explore the connection between the objectives, thus, helping compare the indicators and sort out redundant ones.

The results of applying the NMF does not simply confirm the trivial view that the number of publications and visibility criteria (number of citations) are essential to yield a better ranking, but also establishes some objectives ranked in reverse order, which should be avoided or diminished: publications without DOIs, low male-to-female ratio, short distance cooperation. A recommended publication policy would include complete utilization of open-access possibilities and long-distance collaborations. Naturally, diminishing the female contributions is *not recommended*, *neither is increasing the number of co-authors*. This empirical investigation exposes the conservatism of the academia.

Similarly, the empirical clustering of Asian and Western universities along with the ‘in-between’ does not involve any value judgment but unambiguously shows the different outcome and clustering of the universities. It should also be emphasized that the intermediate position of the top 1% and 5% citations suggest that the role of these indices are over-valued, and they are predominantly determined by random factors. It is reassuring that the analyses return well-known tendencies and commonplaces. The latter reinforces our surprising or uncommon conclusions, make them plausible.

Only coupling of sum of ranking differences and non-negative factorization is able to group the variables indicating cultural differences, at least which are present in the publishing culture. The y axis of Fig 11 is reversed: university goodness grows downwards. Here, it should be emphasized that Leiden and any Web of Science-based assessment down-weights the Asian information circle.

This paper provides a complex analysis of ranking with high dimensionality and *many*-objective optimization. Methods such as principal component analysis (PCA), NMF, and sparse NMF can determine a proper ranking even if the objectives are controversial and rank in reverse. The sum of ranking differences is a handy tool in terms of presampling as it detects unfavourable objectives (random and reversed ranking) that can be later removed.

In university rankings, the grouping of universities by NMF is heavily influenced by national publication policies, namely clusters, depending on where a university belongs in these (inter)national communities. The thorough analysis of the objectives returned trivial statements that the number of publications and citations is a driving force for a higher rank. However, some not-so-trivial results may help to develop an advanced plan for improving publication policy. The endorsement of long-distance and international collaborations is proposed. To promote the latter, an increase in the number of open-access publication may result in many collaborative publications.

By employing NMF for *many*-objective optimization, only a non-negativity constraint needs to be met. The method provides positively correlated objectives and clusters the data (here universities) and the Pareto front. To answer the question of ‘how can ranking be fair?’, we propose the implementation of SRD for filtering and sparse NMF to select universities from throughout the world. Although we discussed the university ranking on an individual dataset, the methodology presented above is entirely general and any dataset that contains meaningful information can be reduced using them. Whereas PCA is hard to interpret, the SRD method orders the objectives, while NMF clusters the objectives, and sparsity removes the weak and redundant objectives from the analysis.

## Supporting information

S1 Appendix Supporting information on TOPSIS. (PDF)

S2 Appendix The interpretation of the principal components. (PDF)

S3 Appendix Mathematical notation. (PDF)

S4 Appendix CWTS Leiden Ranking 2020 database variables. (PDF)
