# Supplementary material for: Matrix factorization-based multi-objective ranking–What makes a good university?
Source: PLoS One. 2023 Apr 13;18(4):e0284078. doi: 10.1371/journal.pone.0284078 (PMC10101413; doi:10.1371/journal.pone.0284078)
Supplement: S1 Appendix — (PDF) [file pone.0284078.s001.pdf]

Matrix factorization-based multi-objective ranking–What makes a good university?

János Abonyi[1*], Ádám Ipkovich[1], Gyula Dörgő[1], Károly Héberger[2]

1 Eötvös Loránd Research Network - University of Pannonia Complex Systems Monitoring Research Group, University of Pannonia, Veszprém, Hungary
2 Plasma Chemistry Research Group, Institute of Materials and Environmental Chemistry, Research Centre for Natural Sciences, Centre of Excellence, Hungarian Academy of Sciences, Budapest
* Corresponding author: janos@abonyilab.com (JA)

S1 Appendix: Supporting information on TOPSIS

TOPSIS [1] is an aggregation-based method that transforms the solutions with many objectives into a single value, whose distance from two extreme points is measured to determine the rank of the solutions. The positive and negative (best and worst) extreme solution can be chosen as $max(f)$ and $min(f)$. TOPSIS ranks according to the best and worst values of the data matrix. TOPSIS is a multi-criteria analysis method as it aggregates multiple conflicting objectives.

First, the input matrix $\mathbf{X}$ is to be normalized.

$$f_k(\mathbf{x}) = \frac{x_{ik}}{\sqrt{\sum_{j=1}^{N} x_{jk}^2}}, i = 1,2,3\dots N, k = 1,2,3\dots n \tag{1}$$

Then the extreme points are determined, and the Euclidean distance between them is calculated for each solution:

$$\rho_{k-} = min(f_1(\mathbf{x}), f_2(\mathbf{x}), \dots, f_n(\mathbf{x})) \tag{2}$$

$$\rho_{k+} = max(f_1(\mathbf{x}), f_2(\mathbf{x}), \dots, f_n(\mathbf{x})) \tag{3}$$

$$\mathbf{d}_{i-} = \sqrt{\sum_{k=1}^{n}(x'_{ik} - \rho_{k-})^2} \tag{4}$$

$$\mathbf{d}_{i+} = \sqrt{\sum_{k=1}^{n}(x'_{ik} - \rho_{k+})^2} \tag{5}$$

where $i = 1, \dots N$ and $k = 1,2,3\dots n$

Next, the distances are scaled between $(0;1)$:

$$\mathbf{s}_{i-} = \frac{\mathbf{d}_{i-}}{(\mathbf{d}_{i-} + \mathbf{d}_{i+})} \tag{6}$$

$$\mathbf{s}_{i+} = \frac{\mathbf{d}_{i+}}{(\mathbf{d}_{i-} + \mathbf{d}_{i+})} \tag{7}$$

where $\mathbf{s}_{i+} = 0, \mathbf{s}_{i-} = 1$ is the best solution; $\mathbf{s}_{i+} = 1, \mathbf{s}_{i-} = 0$ is the worst.

In order to compile a ranking based on TOPSIS, $\mathbf{s}_{i+}$ or $\mathbf{s}_{i-}$ is ranked. All objectives contribute towards the final score of the solutions, but the correlation between them cannot be interpreted appropriately. The structure of the data remains unknown. The steps are described in 1 along with SRD.

TOPSIS is applied to the CWTS Leiden Ranking 2020 database, and the result is in 2. TOPSIS ranks according to two ideal points. To examine the relation of the solutions, we calculated the Spearman's rank correlation [2], which is 1. The first ten Universities are from the Anglosphere, as is denoted in the figure by red circles. On the other hand, the closest solutions, which are illustrated by blue solutions, to the least ideal solution are as follows:

1. Shahrood University of Technology
2. North University of China
3. Annamalai University.
4. Malek Ashtar University of Technology
5. Hebei University
6. Xinjiang University
7. Indian Institute of Technology (Indian School of Mines)
8. Indian Institute of Technology Kanpur
9. Yanshan University
10. Urmia University

References

1. Yoon KP, Kim WK. The behavioral TOPSIS. Expert Systems with Applications. 2017;89:266–272.
2. Fieller EC, Hartley HO, Pearson ES. Tests for rank correlation coefficients. IS. Biometrika. 1957;44.

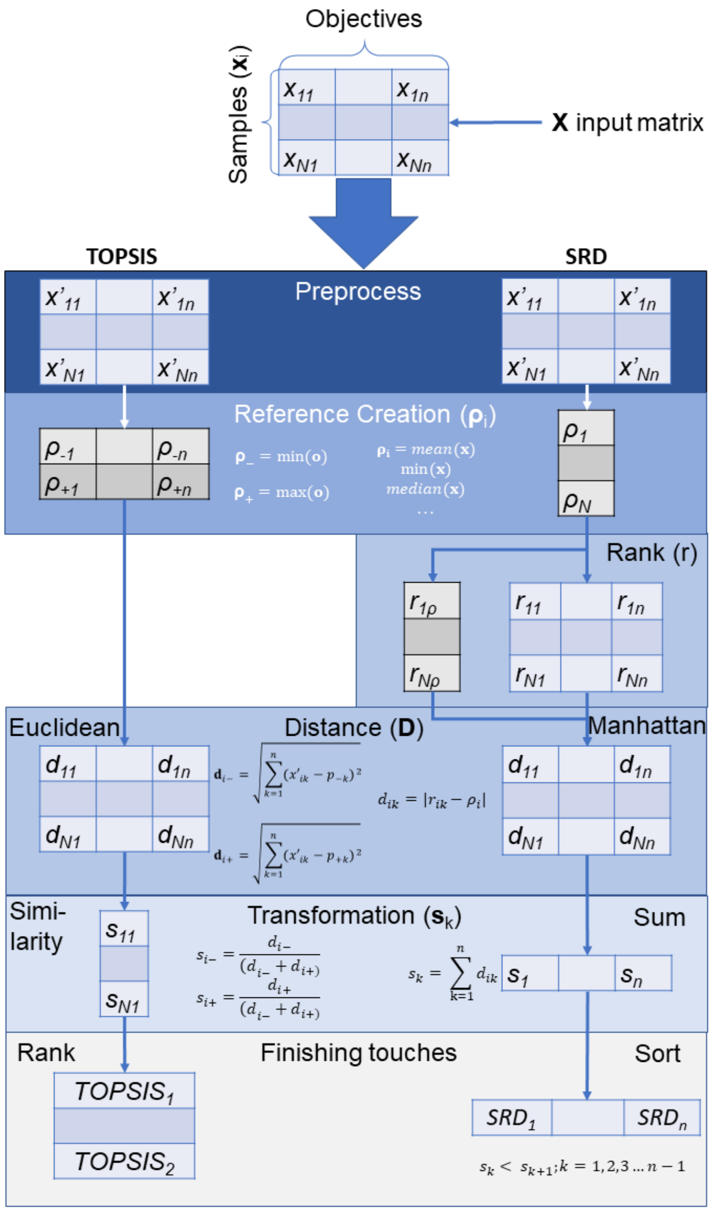

Fig 1. TOPSIS and SRD Flowchart. Although the methods consists of similar steps, they yield fundamentally different results.

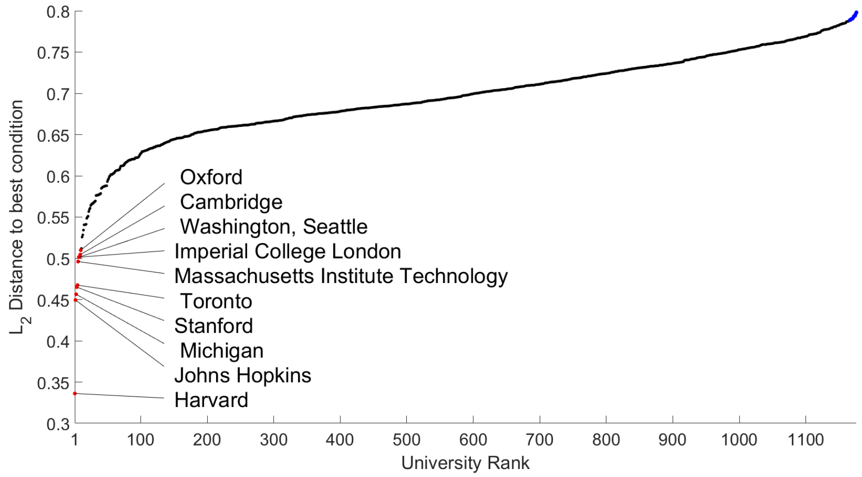

Fig 2. TOPSIS based ranking. The universities are ranked according to their distance to the positive (ideal) solution, *i.e.*, best solution. The red and blue solutions are the top and bottom ten, respectively. The shape drawn by the solutions is almost ideal suggesting high Spearman's rank correlation [2] value and indicates an unknown relationship. Notice that the best universities are in the Anglosphere.
