# Supplementary material for: Matrix factorization-based multi-objective ranking–What makes a good university?
Source: PLoS One. 2023 Apr 13;18(4):e0284078. doi: 10.1371/journal.pone.0284078 (PMC10101413; doi:10.1371/journal.pone.0284078)
Supplement: S2 Appendix — (PDF) [file pone.0284078.s002.pdf]

Matrix factorization-based multi-objective ranking–What makes a good university?

János Abonyi[1*], Ádám Ipkovich[1], Gyula Dörgő[1], Károly Héberger[2]

1 Eötvös Loránd Research Network - University of Pannonia Complex Systems Monitoring Research Group, University of Pannonia, Veszprém, Hungary
2 Plasma Chemistry Research Group, Institute of Materials and Environmental Chemistry, Research Centre for Natural Sciences, Centre of Excellence, Hungarian Academy of Sciences, Budapest
* Corresponding author: janos@abonyilab.com (JA)

S2 Appendix: The interpretation of the principal components

The PCA is extended with the description of the five other PCs and a heatmap of the first seven PCs to provide a better insight to the similarity of the variables. Although not a direct measure of performance, except for the total number of authorships (G1), the gender indicators help establish links between genders and other indicators as well as the relative role of genders amongst current academics. Fig 1 provides information on the principal components. According to PC3 (8.49%), the proportion of female authors (G8, G10) goes hand in hand with the higher number of open-access publications resulting in more frequent collaboration and accessible information. This trend illustrates the modern wave of openness in the traditionally conservative world of the academia. Significant differences exist between the fields that one gender dominates [1]. Although female authors might be more oriented towards people, each profession in a field may yield different proportions in terms of gender, *e.g.*, nurses and neurosurgeons. Male authors tend to focus on abstractions, objects, systems and STEM rather than social calls, education and healthcare. PC4 (5.36%) suggests that the proportion of male authors (G7-G9) correlates with the proportion of industrial collaboration (C9), yet neither genders is significantly related to scientific indicators. On the other hand, in PC5 (4.45%), the proportion of authors of unknown gender correlates with proportional open-access and collaboration indicators, and yet again, no significant connection to scientific indicators is found. PC6 (2.58%) provides information on the correlation between the proportions of long-distance (C11) and international collaboration indicators (C8). PC7 (2.23%) indicates that with regard to the proportion of short-distance collaborations, a weak connection to the proportional scientific indicators and industrial collaboration is observed, as companies often undertake joint projects/researches with universities that promote collaboration and scientific research. The empirical data provided by PC3-5 imply that there is no direct positive connection between scientific impact and gender. The correct indirect correlations are an open-ended question, as a hierarchy of relationships underlies in all the indicators.

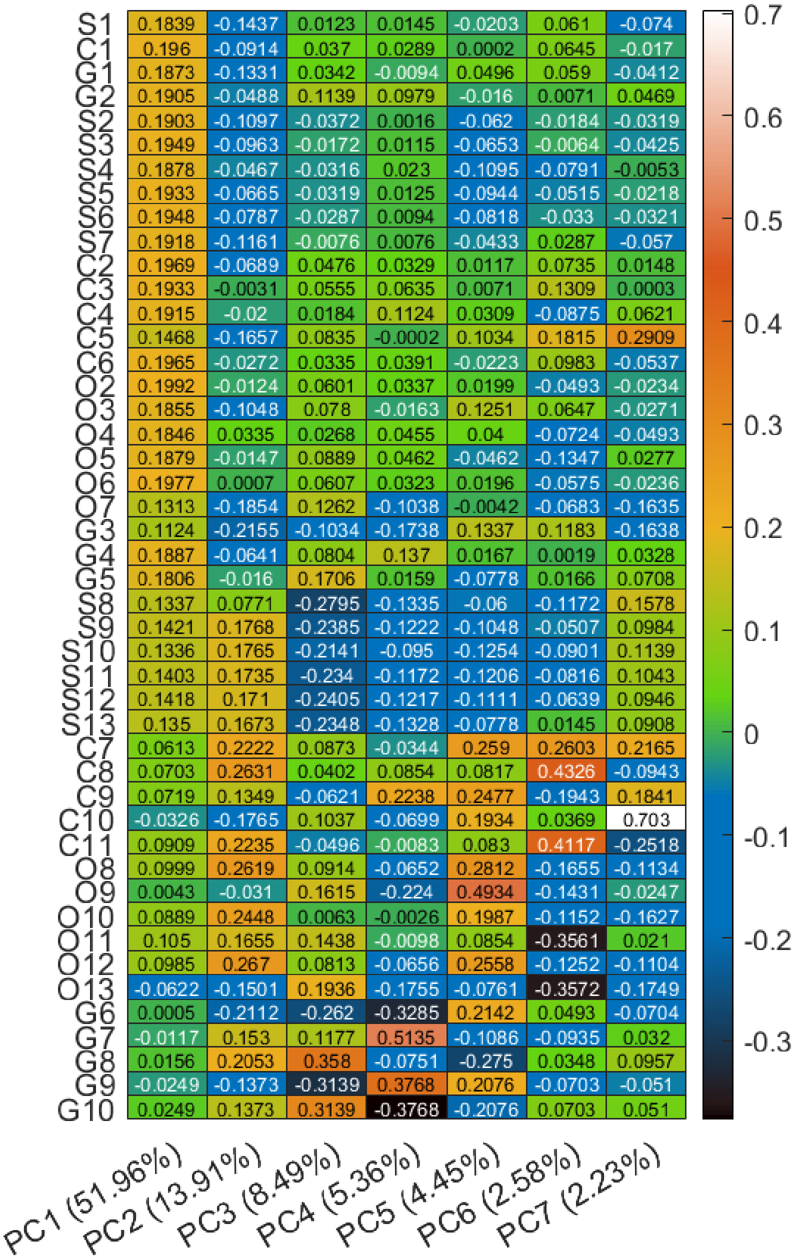

Fig 1. Heatmap of the first seven Principal Component The first PC incorporates most of the quantity indicators, the second incorporates proportional indicators, except for the proportion of unknown gender (G6) and the male to known gender ratio (G9). The third and fourth takes on female and male gender indicators, respectively. The fourth does the same with male indicators. The fifth PC indicates the relationship between open-access and collaboration indicators, while the sixth and seventh mainly features one or two indicators.

References

1. Thelwall M, Bailey C, Tobin C, Bradshaw NA. Gender differences in research areas, methods and topics: Can people and thing orientations explain the results? Journal of Informetrics. 2019;13(1):149–169.
