# Supplementary material for: Matrix factorization-based multi-objective ranking–What makes a good university?
Source: PLoS One. 2023 Apr 13;18(4):e0284078. doi: 10.1371/journal.pone.0284078 (PMC10101413; doi:10.1371/journal.pone.0284078)
Supplement: S3 Appendix — (PDF) [file pone.0284078.s003.pdf]

Matrix factorization-based multi-objective ranking–What makes a good university?

János Abonyi[1*], Ádám Ipkovich[1], Gyula Dörgő[1], Károly Héberger[2]

1 Eötvös Loránd Research Network - University of Pannonia Complex Systems Monitoring Research Group, University of Pannonia, Veszprém, Hungary
2 Plasma Chemistry Research Group, Institute of Materials and Environmental Chemistry, Research Centre for Natural Sciences, Centre of Excellence, Hungarian Academy of Sciences, Budapest
* Corresponding author: janos@abonyilab.com (JA)

S3 Appendix: Mathematical notations

In this appendix, we provide a list of notations used in the publication.

$\mathbf{z}$ - Solution vector

$\mathbf{X}$ - A set of matrix containing the values of the objective functions.

N - Number of solutions (rows).

n - Number of objectives (columns).

$\mathbf{x}$ - A objective function values for a solution, row vector of $\mathbf{X}$, a university.

i, j - Index of a solution, $i, j = 1, 2 \ \dots \ N$.

k - Index of an objective, $k = 1, 2, 3 \dots n$.

$r^{\mathbf{x}}, r^{(}\mathbf{w})$ - Ranks of the objective values of the original and reduced solution respectively, $1, 2, 3 \dots N$.

$f_k(\mathbf{x})$ - The k-th objective function.

$\mathbf{R}$ - Rank(ed) matrix,

$D(r)$ - Number of solutions with rank r.

$RE(D)$ - Relative entropy of an input matrix.

p - Number of objectives after dimensionality reduction, less than n.

$\mathbf{Y}$ - The matrix with reduced dimensionality ($N \times p$).

$g(\mathbf{x}_i)$ - Dimensionality reduction function that reduces an $\mathbf{x}_i$ solution with n amount of objectives to $\mathbf{w}_i$ with p amount of objectives.

$\hat{\mathbf{X}}$ - Reconstructed matrix from the lower-rank approximations.

$\mathbf{W}$ - $N \times p$ representation of the reduced solutions that consists of the reduces solutions.

$\mathbf{H}$ - $p \times n$ coefficient matrix.

α - Regularization parameter to suppress $\mathbf{W}$.

β - Regularization parameter to suppress $\mathbf{H}$.

ρ - Reference vector (*e.g.*, for SRD or TOPSIS). Can be either a column or row vector.

$\mathbf{d}$ - Distance from the respective reference value (ρ). Can be either a column or row vector.

$\mathbf{s}$ - Similarity (in case of TOPSIS), or Sum (in case of SRD). Can be either a column or row vector.
