# Supplementary material for: Matrix factorization-based multi-objective ranking–What makes a good university?
Source: PLoS One. 2023 Apr 13;18(4):e0284078. doi: 10.1371/journal.pone.0284078 (PMC10101413; doi:10.1371/journal.pone.0284078)
Supplement: S4 Appendix — (PDF) [file pone.0284078.s004.pdf]

Matrix factorization-based multi-objective ranking–What makes a good university?

János Abonyi[1*], Ádám Ipkovich[1], Gyula Dörgő[1], Károly Héberger[2]

1 Eötvös Loránd Research Network - University of Pannonia Complex Systems Monitoring Research Group, University of Pannonia, Veszprém, Hungary
2 Plasma Chemistry Research Group, Institute of Materials and Environmental Chemistry, Research Centre for Natural Sciences, Centre of Excellence, Hungarian Academy of Sciences, Budapest
* Corresponding author: janos@abonyilab.com (JA)

S4 Appendix: CWTS Leiden Ranking 2020 database variables

The CWTS Leiden database consists of four major areas: scientific (S), collaboration (C), gender (G) and open-access (O). The areas have several indicators that are referenced with an abbreviated code throughout the article. Thus, a list is presented to name and describe the abbreviations. The first letter is a syllable and denotes the field, while the remaining letters propose the variable, *e.g.*, S7 is the seventh indicators of the scientific discipline - The number of publications that belong to the top 50% most frequently cited ones. The indicators are the following:

S1 - The total number of publications of a university with a scientific impact.

S2, S8 - The total and average numbers of citations of the publications of a university.

S3, S9 - The total and average numbers of citations of the publications of a university, normalized in terms of field and year of publication.

S4, S10 - The number and proportion of a university's publications that, when compared with other publications in the same field and published in the same year, belong to the top 1% most frequently cited ones.

S5, S11 - As described above that belong to the top 5% most frequently cited ones.

S6, S12 - As described above that belong to the top 10% most frequently cited ones.

S7, S13 - As described above that belong to the top 50% most frequently cited ones.

C1 - The total number of publications of a university in collaboration with other universities.

C2, C7 - The number and proportion of a university's publications that have been co-authored with one or more other organizations.

C3, C8 - The number and proportion of a university's publications that two or more countries have co-authored

C4, C9 - The number and proportion of a university's publications that have been co-authored with one or more industrial organizations. All private sector for-profit business enterprises, covering all manufacturing and services sectors, are regarded as industrial organizations.

C5, C10 - The number and proportion of a university's publications with a geographical collaboration distance of less than 100 km.

C6, C11 - The number and proportion of a university's publications with a geographical collaboration distance of more than 5000 km.

G1 - The total number of authorships of a university. For instance, a publication with five authors, of which three and two are reported to be affiliated with university 1 and 2, respectively.

G2 - The number of male and female authorships of a university, that is, a university's number of authorships for which the gender is known.

G3, G6 - The number and proportion (with regard to G1) of authorships of a university for which the gender is unknown.

G4, G7 - The number and proportion (with regard to G1) of male authorships of a university.

G5, G8 - The number and proportion (with regard to G1) of female authorships of a university.

G6 - The number of authorships for which the gender is unknown as a proportion of a university's total number of authorships.

G9 - The number of male authorships as a proportion (with regard to G2) of a university's number of known male and female authorship.

G10 - The number of female authorships as a proportion (with regard to G2) of a university's number of known male and female authorship.

O1 - Removed, as it consists of the same values as C1.

O2, O8 - The number and proportion of open-access publications of a university.

O3, O9 - The number and proportion of gold open-access publications of a university. Gold open-access publications are publications in an open-access journal.

O4, O10 - The number and proportion of hybrid open-access publications of a university. Hybrid open-access publications are publications in a subscription journal that are open-access.

O5, O11 - The number and proportion of bronze open-access publications of a university. Bronze open-access publications are publications in a journal that is open-access without a license.

O6, O12 - The number and proportion of green open-access publications of a university. Green open-access publications are publications in a journal that are also available in an open-access repository.

O7, O13 - The number and proportion of a university's publications for which the open-access status is unknown. These publications typically do not have a DOI in the Web of Science database.
